# The epidemiology of antibiotic-resistant clinical pathogens in Uganda

**DOI:** 10.7189/jogh.14.04184

**Published:** 2024-08-30

**Authors:** Ritah Namusoosa, Ibrahim Mugerwa, Keneth Iceland Kasozi, Allan Muruta, Grace Najjuka, Winifred D Atuhaire, Susan Nabadda, Henry Mwebesa, Charles Olaro, Isaac Ssewanyana, Aloysious Ssemaganda, Adrian Muwonge

**Affiliations:** 1 Department of National Health Laboratories and Diagnostic Services, National Microbiology Reference Laboratory, Ministry of Health, Kampala, Uganda; 2 Department of National Health Laboratories and Diagnostic Services, Ministry of Health, Kampala, Uganda; 3 Infection Medicine, College of Medicine and Veterinary Medicine, Deanery of Biomedical Sciences, The University of Edinburgh, Edinburgh, UK; 4 School of Medicine, Kabale University, Kabale, Uganda; 5 Department of National Disease Control, Ministry of Health, Kampala, Uganda; 6 Directorate of Curative Services, Ministry of Health, Kampala, Uganda; 7 The Digital One Health Laboratory, Division of Epidemiology, Roslin Institute, The University of Edinburgh, Edinburgh, UK

## Abstract

**Background:**

Antibiotic resistance (ABR) is a global challenge, and its control depends on robust evidence primarily derived from surveillance systems.

**Methods:**

We utilised a national surveillance data set to demonstrate how such evidence can be systematically generated. In doing so, we characterised the ABR profiles of priority clinical pathogens, identified associated factors, and drew inferences on antibiotic usage in Uganda.

**Results:**

Of the 12 262 samples collected between 2019–21, we analysed 9033 with complete metadata. ABR was steadily increasing at a rate of 0.5% per year, with a surge in 2021 and the highest and lowest levels of penicillin and carbapenems detected in the northern (odds ratio (OR) = 2.26; *P* < 0.001) and the northeast (OR = 0.28; *P* < 0.001) regions of Uganda respectively. ABR was commonly observed with *Escherichia coli* (OR = 1.18; *P* < 0.001) and *Klebsiella pneumoniae* (OR = 1.25; *P* < 0.001) among older and male patients (61–70 years old) (OR = 1.88; *P* = 0.005). Multi-drug resistance (MDR) and ABR were disproportionately higher among bloodstream infections than respiratory tract infections and urinary tract infections, often caused by *Acinetobacter baumannii.* Co-occurrence of ABR suggests that cephalosporins such as ceftriaxone are in high use all over Uganda.

**Conclusions:**

ABR is indeed a silent pandemic, and our results suggest it is increasing at 0.5% per year, with a notable surge in 2021 likely due to coronavirus disease 2019 (COVID-19). Of concern, ABR and MDR are mainly associated with bloodstream and surgical wound infections, with a gender and age dimension. However, it is encouraging that carbapenem resistance remains relatively low. Such evidence is critical for contextualising the implementation and evaluation of national action plans.

Antibiotic resistance (ABR) is a phenomenon where bacteria become unresponsive to antibiotics, and globally, it is estimated to cause 1.27 million deaths [1]. This disproportionately affects people living in sub-Saharan Africa, where 27.3 deaths per 100 000 population lose their lives due to hard-to-treat infections [2]. Vulnerable populations, including neonates, infants, and pregnant mothers, bear the highest burden [2–4]. The emergence of ABR is associated with the overuse and or misuse of antibiotics in humans and animals, which has increased in the past two decades [5]. It is projected to increase by 67% in the next decade [6]. Global efforts to combat antimicrobial resistance (AMR) require context-specific national action plans (NAPs) to guide control strategies [7]. The lack of robust evidence to contextualise NAPs in several countries limits their ability to tailor interventions and resources. One of the sources of data to generate such evidence is nationwide surveillance [7]. Countries like Uganda have established sentinel site surveillance systems to generate this critical data resource [8,9].

To robustly guide the implementation of NAPs, we used data from this sentinel surveillance network to generate evidence and demonstrate how such a comprehensive phenotypic AMR surveillance data set can be analysed to shape the control of AMR in Uganda.

## METHODS

### Study design and setting

**Figure 1:**
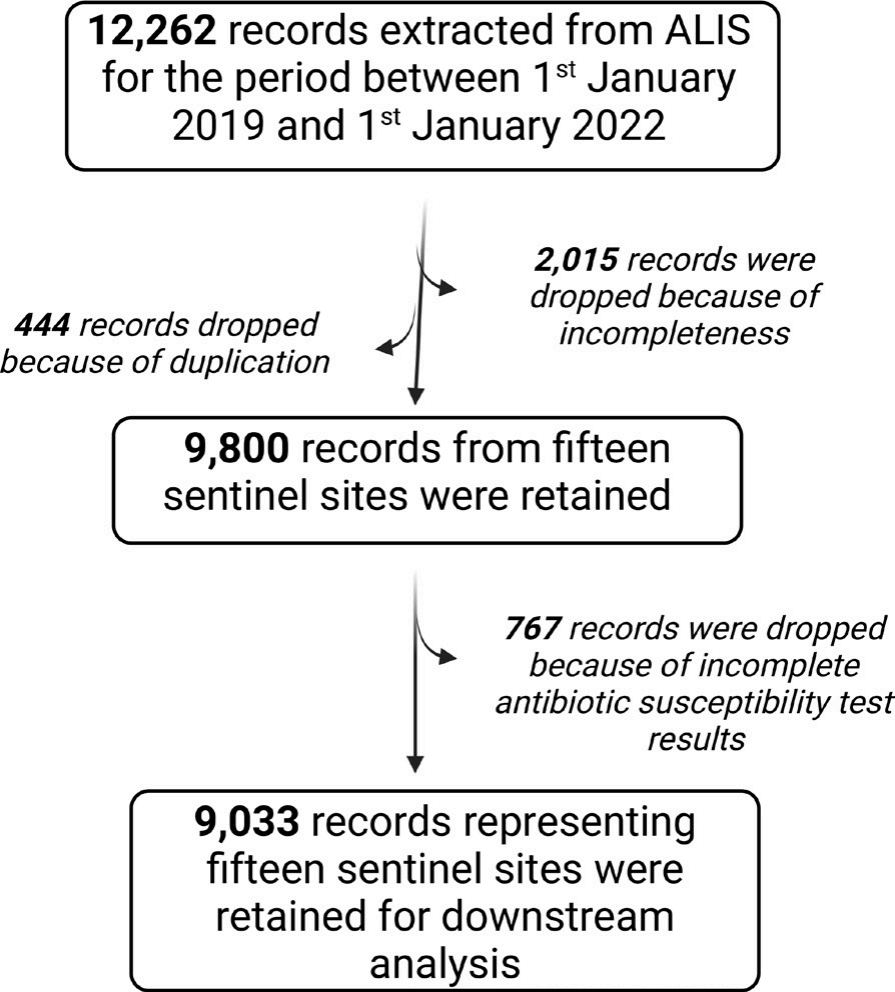
Screening protocol for data sets in the National Laboratory Information system.

In this retrospective analytical study, we used data from the national AMR surveillance collected from a sentinel site surveillance system between 2019–21. The data are collated by the African Laboratory Information System used by the National Microbiology Reference Laboratory at the Ministry of Health in Uganda (Figure S1 in the **Online Supplementary Document**). We did not use patient-identifying variables. We used only variables such as age, sex, district, health facility visited, culture results, and their respective antimicrobial susceptibility test results for monomorphic bacterial organisms. We accessed 12 262 records available in the African Laboratory Information System database. Following data cleaning and deduplication, we included 9033 records from 15 sentinel sites in the analysis (**Figure 1**). Assuming each sample comes from a patient, the sample size is approximately 0.027% of the population. Because over 90% of the population uses government hospitals, this data set provides insights into the clinical characteristics of AMR in Uganda.

### Specimen collection, processing and antimicrobial susceptibility testing

We generated isolates from clinical samples such as blood, urogenital swabs, respiratory samples, aspirates, cerebrospinal fluid, pus, and wound swabs, which were aseptically collected from outpatient and inpatient departments for clinical microbiology [10].

We initially inoculated aspirates, urogenital and wound swabs, respiratory samples, and pus swabs on blood agar, chocolate agar, and MacConkey agar. We subsequently subjected samples showing significant growth to Gram staining. Using calibrated loops, we inoculated urine specimens collected in universal wide-mouthed sterile urine cups on cysteine-lactose-electrolyte deficient agar. Samples with significant growth of ≥10^4^ colony-forming units/ml on cysteine-lactose-electrolyte deficient agar were then subjected to Gram staining. Following isolation, we used the standard biochemical isolation protocols for antimicrobial susceptibility tests (Figure S2 in the **Online Supplementary Document**). We performed antimicrobial susceptibility tests on Mueller Hinton agar using the Kirby-Bauer disc diffusion method, where sensitivity was obtained by measuring the zone diameters of clearance. We selected antibiotic discs, and the results were interpreted following the Clinical Laboratory Standards Institute guidelines. We transported identified culture isolates from the sentinel sites to the National Microbiology Reference Laboratory via the National Sample Transport network for cataloguing and retesting at the National Microbiology Reference Laboratory to confirm the reported phenotype and antimicrobial susceptibility test results. We merged the laboratory-generated data with the metadata for the downstream analysis. Regarding the reliability of the results, the National Microbiology Reference Laboratory is accredited by the College of American Pathologists. It is part of the 3000 laboratories whose quality management systems are monitored by the College of American Pathologists. Regarding the validity of results, the African Laboratory Information System laboratory results are based on retesting isolates from the surveillance system using more harmonised, standard, and automated methods such as Becton Dickinson BACTEC.

### Statistical analysis

We analysed an anonymised data set exported from the African Laboratory Information System (Table S1 in the **Online Supplementary Document**). Therefore, our initial investigation aimed to assess the impact of the coronavirus disease 2019 (COVID-19) pandemic on the national AMR surveillance system. To address this, we compared the trends in culture results with the cumulative burden of COVID-19 cases during the same timeframe. Furthermore, we analysed the incidence of gram-negative and gram-positive pathogens reported per 100 000 population, categorised into quarterly intervals each year.

To visually represent the variation in resistance over time, we plot the distributions of raw disc diffusion diameters for 25 pathogens on 31 antibiotics over three years. The interpretation of these plots is as follows: if the distribution over time shifts towards the left, this suggests an increasing trend in resistance, whereas a shift towards the right indicates a trend of increasing susceptibility. This was done after categorising the antibiotics into access and watch antibiotics in line with the World Health Organization (WHO) Access, Watch, and Reserve antibiotic classification [11].

We then compared the prevalence of antibiotic-resistant pathogens, including monoresistance and multi-resistance patterns, across variables such as gender, age, location, and clinical syndrome of the patients. This enabled us to identify clusters based on temporal, spatial, and patient-related factors. Our second and third research questions were determining the factors associated with mono-drug and multidrug resistance in Uganda. We run several mixed-effects logistic models on data frame subsets by pathogens (i.e. key gram-positive and negative pathogens); antibiotic classes; and the clinical syndromes from which pathogens are recovered. This approach allowed us to appreciate the differential explanatory power of factors such as gender, age group’s location and the temporal effect. The models are developed and implemented using the ‘lme4’ package in R, version 4.1.1 (R Core Team, Vienna, Austria).

The response variable in our model was the bacteria’s monoresistance status, indicating whether they were resistant or susceptible to a particular antibiotic or the presence of multi-resistance. Multi-resistance was defined as resistance to at least three clinically relevant antibiotics for a given pathogen.

The explanatory variables used in the model are listed in Tables S2–3 in the **Online Supplementary Document**. We considered the random variables and tested associations between the response and explanatory variables and their interactions using likelihood ratio tests with the ‘drop1’ function in the ‘lme4’ package. The model selection process began with a complex model and employed backward stepwise variable selection, concluding with the simplest model based on the Akaike information criteria. We assessed the assumptions such as linearity, multicollinearity, and independence. We evaluated the linearity by examining residuals and fitted values of a variable. Further, we checked multicollinearity using the variance inflation factor, and independence was assessed by comparing the correlation of group means to the effects. We determined the normality of residuals using quantile-quantile plots when applicable. Only variables with significant effects (*P*-value <0.05) were retained in the final model.

Finally, as none of the metadata factors included patient antibiotic use, we relied on the established notional relationship between antimicrobial use and antimicrobial resistance to generate co-occurrence networks per pathogen and syndrome, serving as a proxy for antibiotic use. We constructed a binary matrix (1, 0) where ‘1’ indicated resistance and ‘0’ susceptibility to antibiotics. Subsequently, a network was created using the ‘igraph’ package in R, version 1.5.1, where the node and edge thickness represented the number of antibiotics and the frequency of connections between two antibiotics, respectively. The edge thickness is directly proportional to the frequency of their co-occurrence. Thus, the most frequently used antibiotics for a given pathogen can be inferred.

## RESULTS

### Descriptive summary of the study population

**Figure 2:**
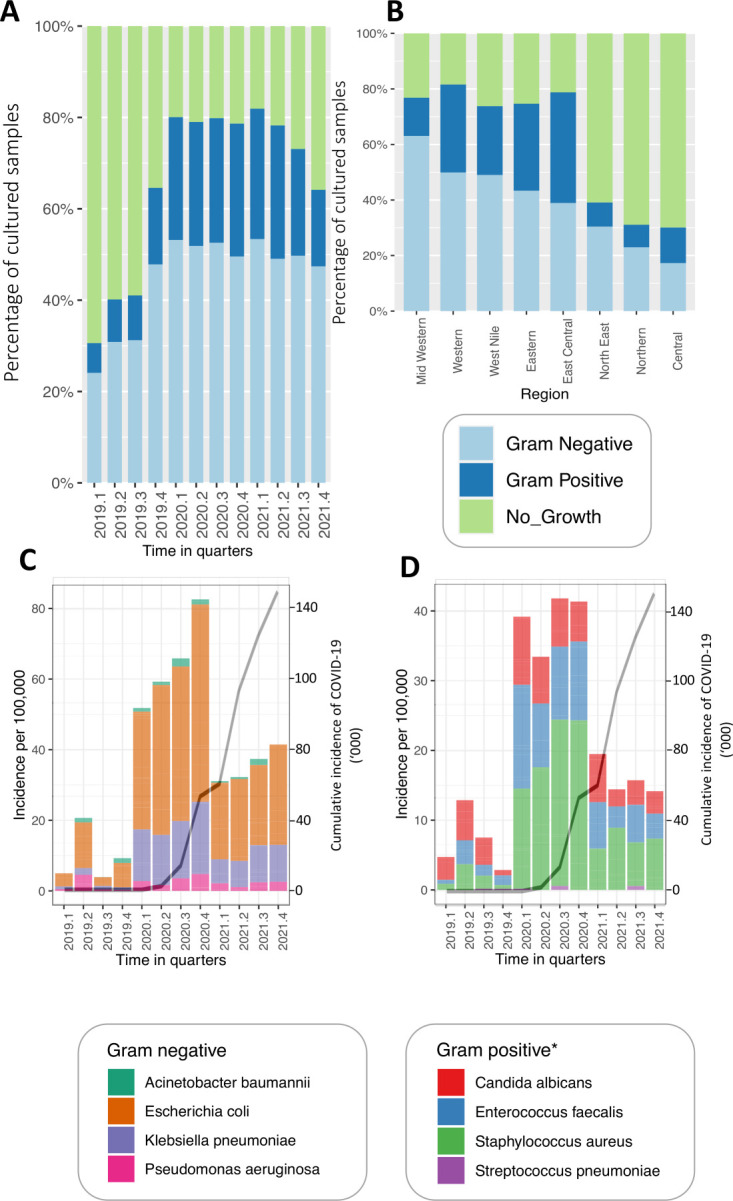
The characteristics and outputs of Uganda’s (ABR) national clinical surveillance system. **Panel A.** The percentage of cultured samples yielded gram-positive and gram-negative bacteria or no growth over the 12 quarters analysed. **Panel B.** The percentage of cultured samples that yielded gram-positive bacteria, gram-negative bacteria, or no growth across the eight regions. **Panel C.** The incidence of gram-negative bacteria across the eight quarters studied. **Panel D.** The incidence of gram-positive bacteria, including yeasts, across the eight quarters studied.

**Figure 4:**
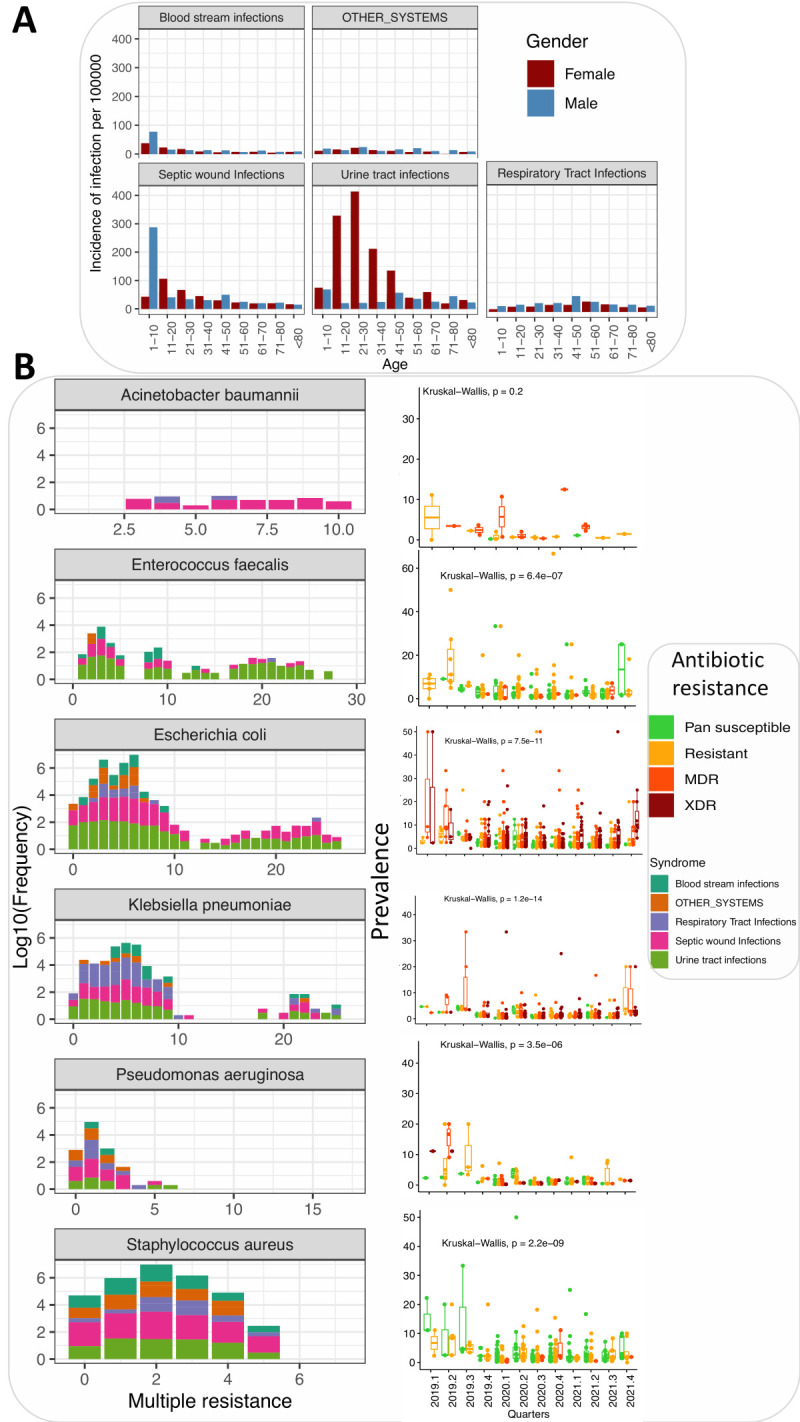
The incidence of clinical syndromes and their relationships with ABR. **Panel A.** The incidence of clinical syndromes by age and gender. It is noteworthy that UTIs were more common among younger females. **Panel B.** Severity of ABR (x-axis represents the number of antibiotics to which the pathogen is resistant) depending on the syndrome. The second panel shows how this severity has been changing over time. For example, the prevalence of extremely resistant (more than five antibiotics) *Escherichia coli*, *Klebsiella pneumoniae*, and *Acinetobacter baumannii* has increased during this study period.

Of 12 262 samples submitted, we analysed 9033 (**Figure 4**, Panel A, Figure S3 in the **Online Supplementary Document**). Of these, 57.8% were females, and their ages ranged from one to 104 years. These yielded 6284 bacteria and 738 yeast, representing a recovery rate of 72.7%, varying across the three years (**Figure 2**, Panel A, Table S1 in the **Online Supplementary Document**) and regions, i.e. the highest and lowest in western and west Nile regions respectively (**Figure 2**, Panel B, Table S1 in the **Online Supplementary Document**). A comparison with COVID-19 incidence showed a reduction in surveillance metrics earlier in the pandemic (**Figure 2****,** Panels C–D).

### ABR patterns of clinical pathogens

Multi-drug resistance (MDR) was more common for *Acinetobacter baumannii* (**Figure 4****,** Panel B) and was predominantly isolated from septic wound infections (SWIs) samples. Mono-resistance was common among *Pseudomonas aeruginosa*, *Escherichia coli*, *Klebsiella. pneumoniae*, and *Staphylococcus aureus* mainly recovered from SWIs and urinary tract infections (UTIs). We also note an increasing frequency of MDR and extensive drug resistance for *Acinetobacter baumannii*, *Escherichia coli*, and *Klebsiella pneumonia*, but less frequent for *Staphylococcus aureus* and *Enterococcus faecalis.*

### Factors associated with clinical ABR

**Figure 5:**
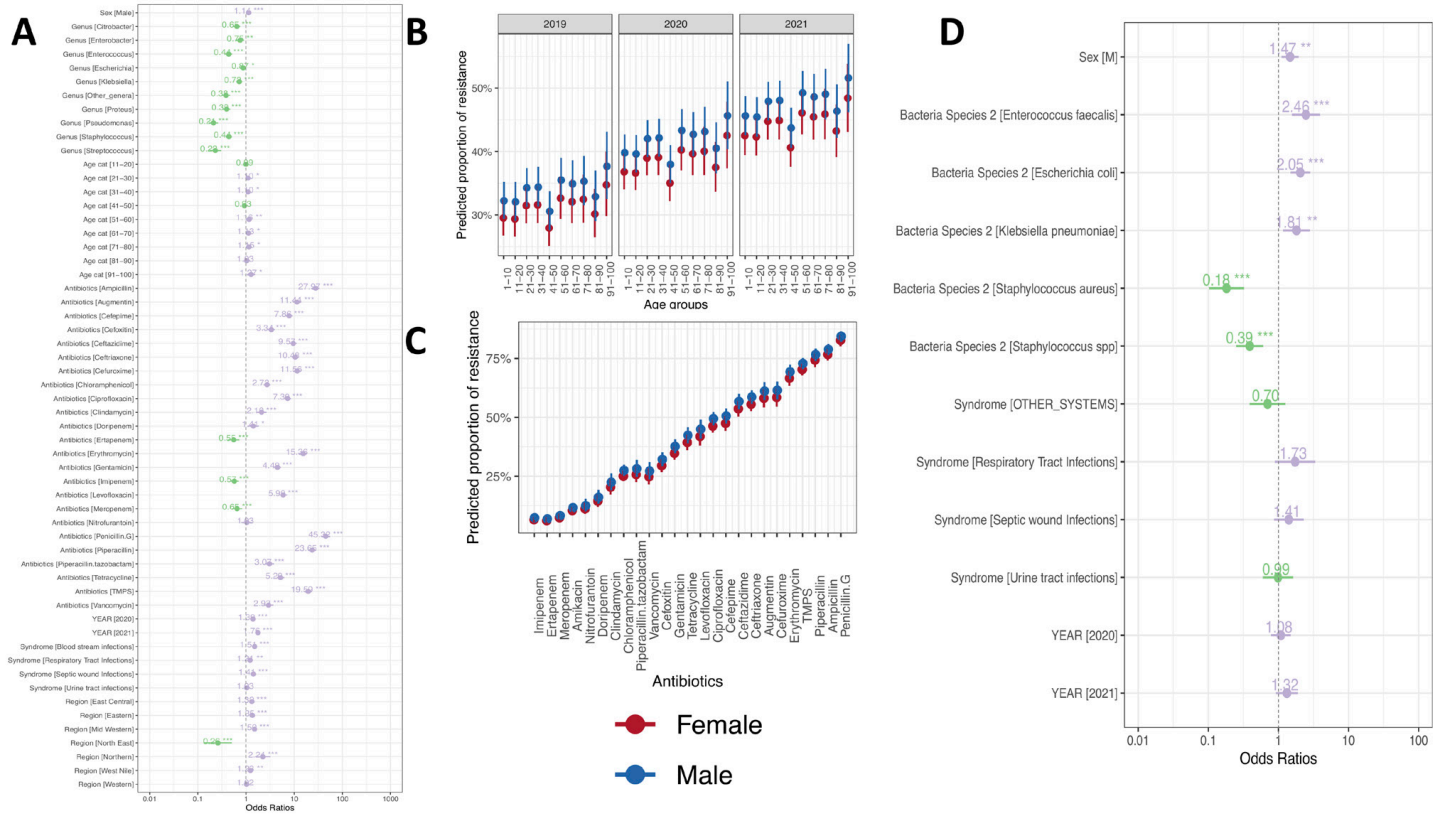
Full mixed effects logistic models examining the drivers of ABR. **Panel A.** The model output for mono and multidrug resistance. **Panel B.** The steady increase of the model predicted ABR across age groups and genders in the three years. **Panel C.** Ranked estimated prevalence of ABR per antibiotic range from 6–78%. **Panel D.** The predicted ABR per antibiotic.

We assessed the potential drivers of the observed ABR levels using mixed logistic regression models. ABR was significantly higher in the northern region of Uganda (odds ratio (OR) = 2.26; 95% confidence interval (CI) = 2.10–2.80, *P* < 0.001) and lowest in the northeast region (OR = 0.28; 95% CI = 0.23–0.31, *P* < 0.001 (**Figure 5**, Panel A). We also showed significant variability elsewhere, with lower levels in the central region relative to eastern (OR = 1.26; 95% CI = 1.30–1.32, *P* < 0.001), mid-western (OR = 1.56; 95% CI = 1.10–1.61, *P* < 0.001) and west Nile (OR = 1.24; 95% CI = 1.12–1.43, *P* = 0.001) (Figure S4, Tables S2–7 in the **Online Supplementary Document**).

### Temporal characteristics of clinical ABR

**Figure 3:**
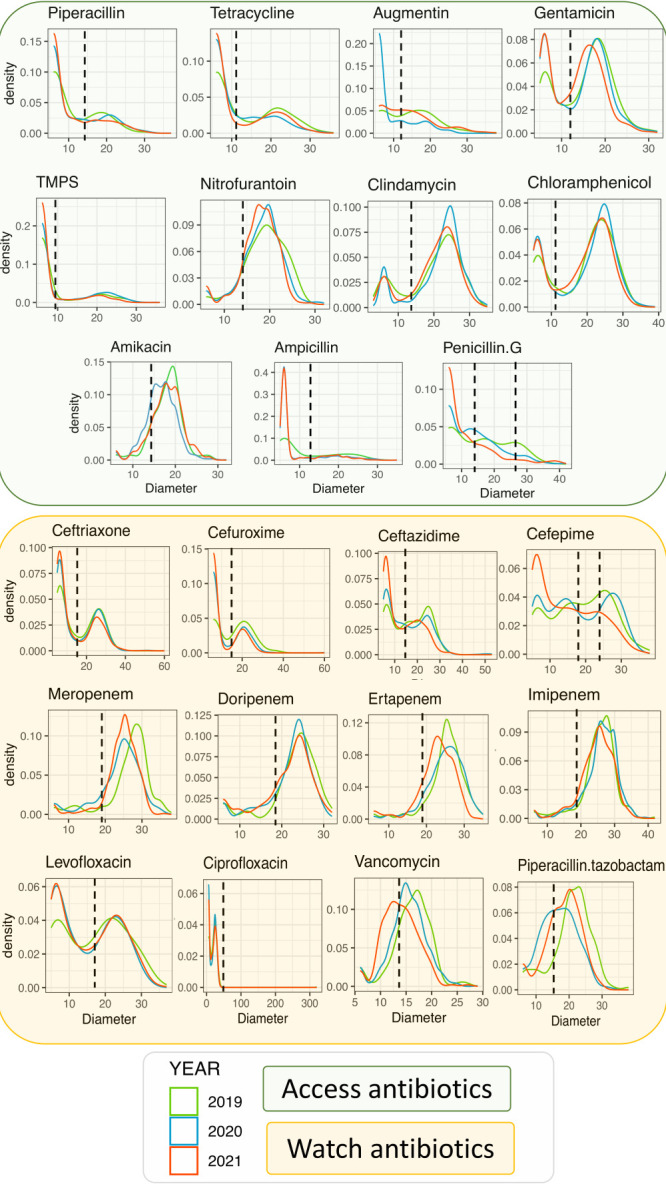
The change in the distribution of disc diffusion diameter measurements over time for eleven and twelve access and reserve antibiotics, respectively. The dotted line shows the threshold for resistance, revealing how much ABR has increased during the study period.

A comparison of disc diffusion diameters of commonly used antibiotics showed a temporal shift in ABR levels (**Figure 3**). For example, we noted an overall shift to the left (increasing resistance) for ceftazidime cefepime, meropenem, ertapenem and vancomycin. Our model showed a consistent increase of ABR to the common antibiotics, with a surge in 2021 (OR = 1.76; *P* < 0.001). For example, an increase in resistance to cephalosporins (OR = 2.27; 95% CI = 1.88–2.75, *P* < 0.001) and tetracycline (OR = 1.73; 95% CI = 1.07–2.79, *P* = 0.024) (**Figure 5**, Panel A and Table S5 in the **Online Supplementary Document**). We also noted a potential seasonality association with ABR, mainly associated with the respiratory tract in the second quarter of each year (OR = 2.18; 95% CI = 1.65–2.90, *P* < 0.001. Generally, we noted a 0.5% increase in resistance each year (Figure S7 in the **Online Supplementary Document**).

### The gender dimension of ABR

The age and gender of patients were associated with ABR; however, this varied depending on other factors such as the syndrome, pathogen, and antibiotic class (**Figure 5**, Panel A). Male patients were more likely to carry ABR pathogen than females, especially if the pathogen in question was *Escherichia coli* (OR = 1.18; 95% CI = 1.12–1.43, *P* < 0.001) or *Klebsiella pneumoniae* (OR = 1.25; 95% CI = 1.12–1.43, *P* < 0.001) isolated from UTIs samples (OR = 1.36; 95% CI = 1.12–1.43, *P* < 0.001) and respiratory tract infections (RTIs) (OR = 1.53; 95% CI = 1.12–1.43, *P* < 0.001). This association was also evident if the antibiotic in question was a fluoroquinolone (OR = 1.28; 95% CI = 1.12–1.43, *P* < 0.001) or cephalosporin (OR = 1.19; 95% CI = 1.12–1.43, *P* = 0.006) (Tables S5–6 in the **Online Supplementary Document**). This gender difference was also reflected in our final model results with various explanatory variables (OR = 1.14; 95% CI = 1.12–1.43, *P* < 0.001). A similar pattern was observed for MDR (OR = 1.47; 95% CI = 1.12–1.43, *P* = 0.007) (Figure S5, Panels A–B in the **Online Supplementary Document**).

### Patient age-specific associations with ABR

The prevalence of ABR increased with age **(****Figure 4**, Panel A), with a significant increase observed among all age groups except for age groups of 11–20 and 41–50 years (Figure S5, Panel A in the **Online Supplementary Document**). Bloodstream infections (BSI)-associated-ABR were less and more common among the age group of 41–50 years compared to the age group of 1–11 years (Figures S4–6, Table S7 in the **Online Supplementary Document**). RTI-associated ABR was more common among the age group of 31–40 years (OR = 1.93; 95% CI = 1.24–3.00, *P* = 0.003) compared to the age group of 1–11 years and more common among males.

### ABR patterns of common clinical syndrome

We observed that, in general, the prevalence of ABR was lower and higher for pathogens recovered from the RTIs and SWIs for most antibiotics. Carbapenem-resistant *Escherichia coli* and *Klebsiella pneumoniae* were more common with SWIs (OR = 2.40; 95% CI = 1.23–4.56, *P* < 0.001) and RTIs (OR = 1.86; 95% CI = 1.12–1.43, *P* = 0.034) (Figure S4 in the **Online Supplementary Document**).

### Clinical pathogen associations with ABR

ABR prevalence was disproportionately higher for *Acinetobacter baumannii,* but we also noted significant variations across clinical pathogens. For example, RTI-associated ABR was less likely among *Klebsiella pneumoniae* and *Enterococcus faecalis* when compared to *Acinetobacter baumannii* (Figure S4, Table S7 in the **Online Supplementary Document**). The full model showed that *Enterococcus faecalis* (OR = 0.47; 95% CI = 0.40–0.55, *P* < 0.001), *Escherichia coli* (OR = 0.87; 95% CI = 0.77–0.99, *P* < 0.028), *Klebsiella pneumoniae* (OR = 0.76; 95% CI = 0.67–0.86, *P* = 0.001) and *Staphylococcus aureus* (OR = 0.45; 95% CI = 0.39–0.51, *P* < 0.001) were less resistant compared to *Acinetobacter baumannii*. *Acinetobacter baumannii* was more likely to express the MDR phenotype than *Staphylococcus aureus* (OR = 0.18; *P* < 0.001) but less likely than *Enterococcus faecalis* (OR = 2.46; *P* < 0.001), *Escherichia coli* (OR = 2.05; 95% CI = 1.26–3.33; *P* < 0.001), and *Klebsiella pneumoniae* (OR = 1.18; *P* = 0.009) (**Figure 5**, Panel D).

### Level of resistance to antibiotic classes

The highest prevalence of ABR was with penicillin, such as penicillin G and ampicillin (**Figure 5**, Panel A). The full model showed overall resistance against penicillins as a class. In particular, penicillin G was significantly higher than most antibiotics tested (Figures S4–6; Table S6 in the **Online Supplementary Document**). The resistance to β-lactam class antibiotics was higher among the female patients (OR = 1.35; *P* = 0.031) and age group of 51–60 years (OR = 1.65; 95% CI = 1.00–2.71, *P* = 0.049) in 2020 (OR = 5.68; 95% CI = 3.95–8.10, *P* < 0.001) and 2021 (OR = 1.79; 95% CI = 1.25–2.55, *P* = 0.001).

Resistance to cephalosporins was significantly lower among patients aged 11–30 years (OR = 0.80; 95% CI = 0.66–0.97, *P* = 0.024) but more likely among male patients with BSI (OR = 1.19; 95% CI = 1.05–1.34, *P* = 0.006). The levels of UTI-related resistance to individual generations of cephalosporins, such as Cefepime and Ceftriaxone, were significantly higher than aminoglycosides, such as amikacin (Table S6 in the **Online Supplementary Document**).

Resistance to macrolides, such as erythromycins, was more common among patients aged 21–30 years (OR = 1.18; 95% CI = 1.24–2.63, *P* = 0.002) and 71–80 years (OR = 2.02; 95% CI = 1.22–3.32, *P* = 0.006) with SWIs (OR = 1.55; 95% CI = 0.37–0.81, *P* = 0.003). Similarly, carbapenem resistance was associated with RTIs (OR = 1.86; 95% CI = 1.05–3.30, *P* = 0.034). On the other hand, fluoroquinolone resistance was not associated with UTIs but with SWIs (OR = 1.56; 95% CI = 1.19–2.05, *P* = 0.001). Finally, resistance to tetracycline was more common among patients with BSI (OR = 3.21; 95% CI = 1.17–8.31, *P* = 0.023), SWI (OR = 3.68; 95% CI = 1.87–7.25, *P* < 0.001) and UTI (OR = 4.11; 95% CI = 2.06–8.20, *P* < 0.001) (Table S6 in the **Online Supplementary Document**).

### Inferring antibiotic use patterns from ABR co-occurrence networks

**Figure 6:**
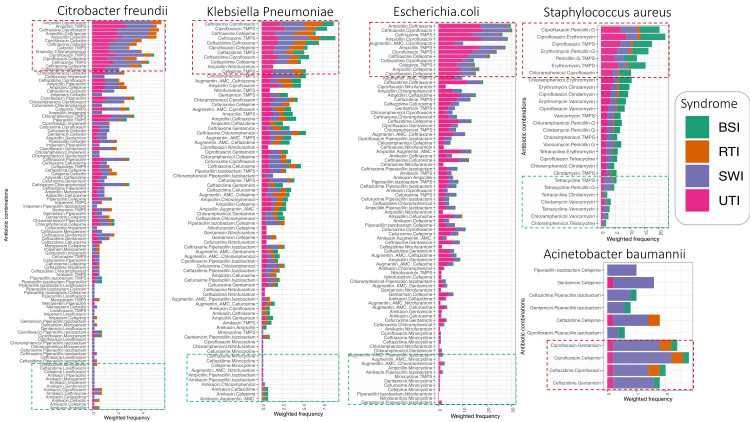
ABR co-occurrence patterns infer antibiotic use patterns among selected clinical bacteria pathogens. The x-axis shows the co-occurrence patterns of the antibiotic, and the y-axis shows the weighted frequency. These are filled based on the syndrome, showing the syndrome a given co-occurrence pattern is associated with. The dotted annotations show the topmost and least frequent pairs.

The ABR co-occurrence patterns among *Acinetobacter baumannii* strains suggested using a profile that includes ciprofloxacin, cefepime, gentamicin, and ceftazidime to manage SWIs. For *Escherichia coli* and *Klebsiella pneumoniae*, the results suggested a difference in antibiotic use to manage SWIs and UTIs (**Figure 6**). Antibiotic use overlapped considerably, including ceftriaxone, ciprofloxacin, ampicillin, trimethoprim-sulfamethoxazole (TMP), and ceftazidime across syndromes. The inferred patterns for gram-positive pathogens such as *Staphylococcus aureus* revealed associated clinical syndromes and potential use of penicillin G, erythromycin, ciprofloxacin and TMP.

## DISCUSSION

We analysed Uganda’s antibiotic resistance surveillance database between 2019–21 to provide evidence of the clinical ABR burden and inform contextualisation of the national action plans [12]. Our findings revealed a steady increase in the prevalence of ABR clinical pathogens, with a notable surge in the last year. We also highlighted the potential impact of COVID-19 on the output of the national AMR surveillance system [13]. The findings showed how integral age and gender are to the prevalence of ABR among pathogens recovered from common clinical syndromes. The highest and lowest resistance was observed against penicillin, such as ampicillin, penicillin G, and carbapenems, respectively. This evidence highlights a complex and multi-level risk landscape, which we discuss in detail below.

### COVID-19 response disrupted ABR surveillance

We observed both quantitative (number of samples processed) and qualitative (key performance indicators) reductions in the performance of the AMR National Sentinel Site Surveillance system at the Ministry of Health. The decline in performance coincided with the COVID-19 lockdown in the second quarter of 2020, indicating a plausible connection to increased demands on the health care system and a likely reallocation of funding and human resources. As reported elsewhere, the decrease in key performance indicators, such as culture recovery rate, may reflect competing priorities associated with the pandemic period [14,15]. Interestingly, the decline in qualitative and quantitative performance did not correspond to a decrease in ABR levels. On the contrary, there was an observed increase in ABR levels, notably in 2021, compared to pre-pandemic times. This rise is likely attributable to the increased use of antibiotics for treatment and prophylaxis linked to COVID-19 [16,17]. It is evident that syndromes such as RTIs, SWIs, and UTIs commence in the second quarter of each year and continue to rise consistently with different rates per syndrome. It may also be due to disease season effects associated with the start of the rainy season [18,19]. This suggests that the ABR surge in 2021 might be due to intrinsic seasonal factors and extrinsic selection pressures driven by COVID-19-associated antibiotic use. Indeed, overall, we observe a steady increase in the resistance prevalence at a rate of 0.5% per year (Figure S1 in the **Online Supplementary Document**). Therefore, targeted interventions are required to halt and reverse this trend.

### The gender perspective of clinical ABR in Uganda

Literature on gender-based differences linked to ABR remains limited, but we present evidence that it was higher among females. This was attributed to the higher lifetime use among females than men [20,21]. Here, we found a higher prevalence among male patients, especially for pathogens like *Klebsiella pneumoniae* and *Escherichia coli* associated with RTIs and UTIs. Male patients were more likely to carry MDR pathogens. Considering health-seeking behaviour, this difference may reflect the notion that male patients self-medicate and only visit health centres with syndromes that have progressed, which might also explain the higher MDR. Indeed, recent studies on lower respiratory infections have shown that male patients are 1.31 times more likely to succumb to disease compared to females [19] and more likely to abuse or overuse antibiotics [22].

### Clinical ABR increases with the patient’s age

Our findings agree with the widely accepted notion that lifetime antibiotic exposure directly correlates with ABR [23,24]. Here, we noted a steady increase in ABR across age groups, although the relationship varied based on the syndrome, pathogen, and antibiotic in question. For instance, there is a disproportionately high occurrence of ABR *Klebsiella pneumoniae* among 31–40-year-olds with RTIs. Similarly, there is a higher incidence of cephalosporin, penicillin G, and vancomycin-resistant *Klebsiella pneumoniae* and *Staphylococcus aureus* among 11–30-year-olds with SWIs [25,26]. Tetracycline resistance is often regarded as legacy resistance due to its extensive use over the last 50 years [27]. We found elevated levels of tetracycline resistance among patients aged 51–60, supporting lifetime exposure. Our findings indicate that antibiotic-resistant BSIs was higher among infants and patients aged 61–70 years, likely associated with high levels of antibiotic use to manage such life-threatening syndromes.

### Syndrome-specific variability of ABR among priority clinical pathogens

Here, we focused on eight of 12 WHO priority bacteria pathogens [28], causing syndromes such as UTIs and RTIs, which collectively account for a global burden of 150 million [29] and 17.2 million cases annually [30], respectively. We observed a growing prevalence of ABR associated with *Escherichia coli* and *Klebsiella pneumoniae* linked to these syndromes [31–33]. While UTIs and RTIs were the most prevalent, BSI *Staphylococcus aureus*, *Escherichia coli*, and *Klebsiella pneumoniae* were disproportionately antibiotic-resistant, as reported elsewhere [34]. In Europe, *Staphylococcus aureus* BSI is estimated at 20–30 per 100 000 population [35,36], and low- and middle-income countries are linked to poor prognoses [34,37]. In Uganda, we noted a growing level of vancomycin-resistant *Staphylococcus aureus* and *Enterococcus faecalis* which may exacerbate an already challenging paediatric BSIs [38] and surgical outcomes.

Globally, BSI caused by *Escherichia coli* and *Klebsiella pneumoniae* surpasses that of *Staphylococcus aureus* and is growing amplified by region-specific hypervirulent strains [19]. However, in this study, we observed significantly higher resistance levels to narrow-spectrum antibiotics among *Klebsiella pneumoniae* and *Escherichia coli* isolates during 2020–21. Furthermore, we observed substantial resistance against fluoroquinolones and cephalosporins but comparatively lower resistance against carbapenems. Therefore, it is imperative for NAPs to emphasise strategies that protect the current utility of carbapenems.

Lastly, we noted high levels of ABR and MDR associated with SWIs caused by *Acinetobacter baumannii*. Anecdotal reports show that this pathogen accounts for nearly 18% of all SWIs cases at Mulago National Referral Hospital [39], where it was characterised as a ‘difficult to treat’ pathogen because of its multidrug resistance phenotype, including carbapenem-resistant *Acinetobacter baumannii* [40]. Indeed, the success of this opportunistic clinical pathogen is extensively documented in the literature [41,42]. Therefore, the observed characteristics are likely due to its diverse resistance mechanisms, including β-lactamase acquisition, aminoglycoside modification, permeability defects, up-regulation of multidrug efflux pumps, and alterations in target sites [43].

### ABR co-occurrence could be used to infer antibiotic use

In this analysis, we exploited the empirical relationship between antimicrobial use and ABR [44,45] so the ABR co-occurrence networks can infer potential antibiotic use in clinical and community settings. We inferred antimicrobial use for eleven molecules, namely ceftriaxone, cefoxitin, cefepime, ceftazidime, ciprofloxacin, ampicillin, and TMP, which likely dominate the management of clinical pathogens such as *Escherichia coli*, *Klebsiella pneumoniae*, *Acinetobacter baumannii*, and *Citrobacter freundii* in Uganda. Likely, their use for *Acinetobacter baumannii* is almost exclusively linked to the management of SWIs. Based on our findings, ceftriaxone is probably the most used antibiotic to manage BSIs, UTIs and RTIs. Indeed, this is the case in most clinical settings, where the next option is usually cefixime. For gram-positive pathogens such as *Staphylococcus aureus,* treatment is likely dominated by penicillin G, erythromycin, ciprofloxacin, chloramphenicol, TMP, and vancomycin. Remarkably, our inferences closely aligned with the treatment guidelines outlined in the Uganda clinical guidelines from 2016 [46].

### Study limitations

Some districts may be underrepresented based on their respective regional referral hospital laboratories’ capacity to submit samples to the central referral laboratory. We acknowledge that this may affect the generalisability of this data at the national level. We have overcome this problem by clustering the district into regions, allowing sufficient degrees of freedom for the analysis. It is also noteworthy that most of the isolates analysed were from government facilities, with less or few from the private health sector. Therefore, this clinical picture is limited to government facilities. To fully map these dynamics, we need to combine government and private sector data.

## CONCLUSIONS

Our results indicated a steady increase in ABR of approximately 0.5% per year, with a notable upsurge in 2021 likely due to imprudent antibiotic usage during the COVID-19 pandemic. SWIs were primarily caused by multi-resistant *Acinetobacter baumannii*, but BSIs were caused by resistant *Staphylococcus aureus*. Methicillin-resistant *Staphylococcus aureus* was associated with SWIs. RTIs and UTIs were mainly caused by *Klebsiella pneumoniae* and *Escherichia coli*, which are resistant to fluoroquinolone and cephalosporins and disproportionately prevalent among male patients. Encouragingly, resistance against carbapenems was relatively low prevalence, underscoring the need to protect this utility.

## Additional material


Online Supplementary Document

## References

[1] Ranjbar R Alam M Antimicrobial Resistance Collaborators 2022 Global burden of bacterial antimicrobial resistance in 2019: a systematic analysis. Evid Based Nurs. 2023:ebnurs-2022-103540. 37500506 10.1136/ebnurs-2022-103540

[2] Taylor J, Hafner M, Yerushalmi E, Smith R, Bellasio J, Vardavas R, et al. Estimating the economic costs of antimicrobial resistance: Model and Results. Cambridge, UK: RAND Corporation; 2014. Available: https://www.rand.org/pubs/research_reports/RR911.html. Accessed: 10 December 2023.

[3] Mayanja R Muwonge A Aruhomukama D Katabazi FA Bbuye M Kigozi E Source-tracking ESBL-producing bacteria at the maternity ward of Mulago hospital, Uganda. PLoS One. 2023;18:e0286955. 10.1371/journal.pone.0286955 37289837 PMC10249850

[4] Jasovský D Littmann J Zorzet A Cars O Antimicrobial Resistance - A Threat to the World’s Sustainable Development - Dag Hammarskjöld Foundation. Ups J Med Sci. 2016;121:159–64. 10.1080/03009734.2016.1195900 27416324 PMC4967260

[5] Browne AJ Chipeta MG Haines-Woodhouse G Kumaran PAE Hamadani BHK Zaraa S Global antibiotic consumption and usage in humans, 2000–18: a spatial modelling study. Lancet Planet Health. 2021;5:e893–904. 10.1016/S2542-5196(21)00280-1 34774223 PMC8654683

[6] Larsson DGJ Flach CF Antibiotic resistance in the environment. Nat Rev Microbiol. 2022;20:257–69. 10.1038/s41579-021-00649-x 34737424 PMC8567979

[7] World Health Organization. Global action plan on antimicrobial resistance. Geneva: World Health Organization; 2015. Available: https://www.who.int/publications/i/item/9789241509763. Accessed: 10 December 2023.

[8] Uganda National Academy of Sciences. Antibiotic Resistance in Uganda: Situation Analysis and Recommendations. Uganda: Uganda National Academy of Sciences; 2015. Available: https://onehealthtrust.org/wp-content/uploads/2017/06/uganda_antibiotic_resistance_situation_reportgarp_uganda_0-1.pdf. Accessed: 13 June 2023.

[9] Ministry of Health, National Action Plans and Monitoring and Evaluation. Uganda: Antimicrobial Resistance National Action Plan 2018-2023. Uganda: Ministry of Health; 2018. Available: https://www.who.int/publications/m/item/uganda-antimicrobial-resistance-national-action-plan-2018-2023. Accessed: 17 July 2023.

[10] Uganda Ministry of Health. National Microbiology Reference Laboratory. Laboratory Handbook. Uganda: Ministry of Health; 2020. Available: https://www.nicd.ac.za/wp-content/uploads/2020/11/NICD-Laboratory-Handbook_V18.pdf. Accessed: 21 August 2024.

[11] Zanichelli V Sharland M Cappello B Moja L Getahun H Pessoa-Silva C The WHO AWaRe (Access, Watch, Reserve) antibiotic book and prevention of antimicrobial resistance. Bull World Health Organ. 2023;101:290–6. 10.2471/BLT.22.288614

[12] Mugerwa I Nabadda SN Midega J Guma C Kalyesubula S Muwonge A Antimicrobial Resistance Situational Analysis 2019–2020: Design and Performance for Human Health Surveillance in Uganda. Trop Med Infect Dis. 2021;6:178. 10.3390/tropicalmed6040178 34698282 PMC8544686

[13] Tomczyk S Taylor A Brown A de Kraker MEA El-Saed A Alshamrani M Impact of the COVID-19 pandemic on the surveillance, prevention and control of antimicrobial resistance: A global survey. J Antimicrob Chemother. 2021;76:3045–58. 10.1093/jac/dkab300 34473285 PMC8499888

[14] Frawley T van Gelderen F Somanadhan S Coveney K Phelan A Lynam-Loane P The impact of COVID-19 on health systems, mental health and the potential for nursing. Ir J Psychol Med. 2021;38:220–6. 10.1017/ipm.2020.105 32933594 PMC7596574

[15] Haileamlak A The impact of COVID-19 on health and health systems. Ethiop J Health Sci. 2021;31:1073–4. 35392335 10.4314/ejhs.v31i6.1 PMC8968362

[16] Dare SS Eze ED Echoru I Usman IM Ssempijja F Bukenya EE Behavioural Response To Self-Medication Practice Before and During Covid-19 Pandemic in Western Uganda. Patient Prefer Adherence. 2022;16:2247–57. 10.2147/PPA.S370954 36034331 PMC9400814

[17] Olamijuwon E Keenan K Mushi MF Kansiime C Konje ET Kesby M Treatment seeking and antibiotic use for urinary tract infection symptoms in the time of COVID-19 in Tanzania and Uganda. J Glob Health. 2024;14:05007. 10.7189/jogh.14.05007 38236690 PMC10795859

[18] Rutebemberwa E Mpeka B Pariyo G Peterson S Mworozi E Bwanga F High prevalence of antibiotic resistance in nasopharyngeal bacterial isolates from healthy children in rural Uganda: A cross-sectional study. Ups J Med Sci. 2015;120:249–56. 10.3109/03009734.2015.1072606 26305429 PMC4816885

[19] Muwanguzi TE Yadesa TM Agaba AG Antibacterial prescription and the associated factors among outpatients diagnosed with respiratory tract infections in Mbarara Municipality, Uganda. BMC Pulm Med. 2021;21:374. 10.1186/s12890-021-01739-5 34781920 PMC8591439

[20] Depshikha B, Srishti G. Antimicrobial Resistance and Gender - One Health Trust. 2022. Available: https://onehealthtrust.org/news-media/blog/antimicrobial-resistance-and-gender/. Accessed: 1 August 2023.

[21] Wong C Drug-resistant infections more likely to strike women, says WHO. Nature. 2024. Online ahead of print. 10.1038/d41586-024-01476-9 38858551

[22] Santella B Serretiello E De Filippis A Veronica F Iervolino D Dell’Annunziata F Lower Respiratory Tract Pathogens and Their Antimicrobial Susceptibility Pattern: A 5-Year Study. Antibiotics (Basel). 2021;10:851. 10.3390/antibiotics10070851 34356772 PMC8300710

[23] Abejew AA Wubetu GY Fenta TG Relationship between Antibiotic Consumption and Resistance: A Systematic Review. Can J Infect Dis Med Microbiol. 2024;2024:9958678. 10.1155/2024/9958678 38476862 PMC10932619

[24] Todman H Arya S Baker M Stekel DJ A model of antibiotic resistance genes accumulation through lifetime exposure from food intake and antibiotic treatment. PLoS One. 2023;18:e0289941. 10.1371/journal.pone.0289941 37590256 PMC10434901

[25] Ntirenganya C Muvunyi CM Manzi O Ogbuagu O High Prevalence of Antimicrobial Resistance Among Common Bacterial Isolates in a Tertiary Healthcare Facility in Rwanda. Am J Trop Med Hyg. 2015;92:865–70. 10.4269/ajtmh.14-0607 25646259 PMC4385787

[26] Mancuso G Midiri A Gerace E Biondo C Bacterial Antibiotic Resistance: The Most Critical Pathogens. Pathogens. 2021;10:1310. 10.3390/pathogens10101310 34684258 PMC8541462

[27] Grossman TH Tetracycline Antibiotics and Resistance. Cold Spring Harb Perspect Med. 2016;6:a025387. 10.1101/cshperspect.a025387 26989065 PMC4817740

[28] World Health Organization. WHO bacterial priority pathogens list, 2024: Bacterial pathogens of public health importance to guide research, development and strategies to prevent and control antimicrobial resistance. Geneva: World Health Organization; 2024. Available: https://www.who.int/publications/i/item/9789240093461. Accessed: 13 January 2024.

[29] Zeng Z Zhan J Zhang K Chen H Cheng S Global, regional, and national burden of urinary tract infections from 1990 to 2019: an analysis of the global burden of disease study 2019. World J Urol. 2022;40:755–63. 10.1007/s00345-021-03913-0 35066637

[30] Jin X Ren J Li R Gao L Zhang H Li J Global burden of upper respiratory infections in 204 countries and territories, from 1990 to 2019. EClinicalMedicine. 2021;37:100986. 10.1016/j.eclinm.2021.100986 34386754 PMC8343248

[31] Zhou Y Zhou Z Zheng L Gong Z Li Y Jin Y Urinary Tract Infections Caused by Uropathogenic Escherichia coli: Mechanisms of Infection and Treatment Options. Int J Mol Sci. 2023;24:10537. 10.3390/ijms241310537 37445714 PMC10341809

[32] Okojie RO Omorokpe VO A survey on urinary tract infection associated with two most common uropathogenic bacteria. Afr J Clin Exp Microbiol. 2018;19:171–6. 10.4314/ajcem.v19i3.3

[33] Jalil MB Al Atbee MYN The prevalence of multiple drug resistance Escherichia coli and Klebsiella pneumoniae isolated from patients with urinary tract infections. J Clin Lab Anal. 2022;36:e24619. 10.1002/jcla.24619 35870190 PMC9459318

[34] Allel K Stone J Undurraga EA Day L Moore CE Lin L The impact of inpatient bloodstream infections caused by antibiotic-resistant bacteria in low- and middle-income countries: A systematic review and meta-analysis. PLoS Med. 2023;20:e1004199. 10.1371/journal.pmed.1004199 37347726 PMC10287017

[35] Kimmig A Hagel S Weis S Bahrs C Löffler B Pletz MW Management of Staphylococcus aureus Bloodstream Infections. Front Med (Lausanne). 2021;7:616524. 10.3389/fmed.2020.616524 33748151 PMC7973019

[36] Kern WV Rieg S Burden of bacterial bloodstream infection-a brief update on epidemiology and significance of multidrug-resistant pathogens. Clin Microbiol Infect. 2020;26:151–7. 10.1016/j.cmi.2019.10.031 31712069

[37] Antimicrobial Resistance Collaborators Global burden of bacterial antimicrobial resistance in 2019: a systematic analysis. Lancet. 2022;399:629–55. 10.1016/S0140-6736(21)02724-0 35065702 PMC8841637

[38] Carrasco Calzada F Jairo Aguilera J Moreno JE Cuadros González J Roca Biosca D Prieto-Pérez L Differences in Virulence Factors and Antimicrobial Susceptibility of Uropathogenic Enterococcus spp. Strains in a Rural Area of Uganda and a Spanish Secondary Hospital. Trop Med Infect Dis. 2023;8:282. 10.3390/tropicalmed8050282 37235330 PMC10223631

[39] Mboowa G Aruhomukama D Sserwadda I Kitutu FE Davtyan H Owiti P Increasing antimicrobial resistance in surgical wards at mulago national referral hospital, uganda, from 2014 to 2018-cause for concern? Trop Med Infect Dis. 2021;6:82. 10.3390/tropicalmed6020082 34069345 PMC8163195

[40] Shields RK Paterson DL Tamma PD Navigating Available Treatment Options for Carbapenem-Resistant Acinetobacter baumannii-calcoaceticus Complex Infections. Clin Infect Dis. 2023;76:S179–93. 10.1093/cid/ciad094 37125467 PMC10150276

[41] Arowolo MT Orababa OQ Olaitan MO Osibeluwo BV Essiet UU Batholomew OH Prevalence of carbapenem resistance in Acinetobacter baumannii and Pseudomonas aeruginosa in sub-Saharan Africa: A systematic review and meta-analysis. PLoS One. 2023;18:e0287762. 10.1371/journal.pone.0287762 38015906 PMC10684001

[42] Kipsang F Munyiva J Menza N Musyoki A Carbapenem-resistant Acinetobacter baumannii infections: Antimicrobial resistance patterns and risk factors for acquisition in a Kenyan intensive care unit. IJID Reg. 2023;9:111–6. 10.1016/j.ijregi.2023.10.007 38020185 PMC10652105

[43] Nasrollahian S Graham JP Halaji M A review of the mechanisms that confer antibiotic resistance in pathotypes of E. coli. Front Cell Infect Microbiol. 2024;14:1387497. 10.3389/fcimb.2024.1387497 38638826 PMC11024256

[44] Bronzwaer SLAM Cars O Buchholz U Mölstad S Goettsch W Veldhuijzen IK A European study on the relationship between antimicrobial use and antimicrobial resistance. Emerg Infect Dis. 2002;8:278–82. 10.3201/eid0803.010192 11927025 PMC2732471

[45] Lipsitch M Samore MH Antimicrobial use and antimicrobial resistance: a population perspective. Emerg Infect Dis. 2002;8:347–54. 10.3201/eid0804.010312 11971765 PMC2730242

[46] Ministry of Health. National guidelines for management of common conditions. Uganda: Ministry of Health; 2016. Available: https://www.prb.org/wp-content/uploads/2018/05/Uganda-Clinical-Guidelines-2016-National-Guidelines-for-Management-of-Common-Conditions.pdf. Accessed: 12 December 2023.

