# Supplementary material for: The epidemiology of antibiotic-resistant clinical pathogens in Uganda
Source: J Glob Health. 2024 Aug 30;14:04184. doi: 10.7189/jogh.14.04184 (PMC11364088; doi:10.7189/jogh.14.04184)
Supplement: Online Supplementary Document [file jogh-14-04184-s001.pdf]

Supplementary Material: **The epidemiology of antibiotic-resistant clinical pathogens in Uganda**

Ritah Namusoosa[1*], Ibrahimm Mugerwa[2], Keneth Iceland Kasozi[3,4*], Allan Muruta[5], Grace Najjuka[1], Winifred. D. Atuhaire[1], Susan Nabadda[2], Henry Mwebesa [6], Olaro Charlse[6], Isaac Ssewanyana[2], Adrian Muwonge[7*]

<u>Affiliations</u>

1. Department of National Health Laboratories and Diagnostic Services, National Microbiology Ref. Laboratory- Ministry of Health, Uganda
2. Department of National Health Laboratories and Diagnostic Services, Ministry of Health, Uganda
3. Infection Medicine, College of Medicine and Veterinary Medicine, Institute for Regeneration and Repair, Edinburgh Bio-Quarter, 4-5 Little France Drive, Edinburgh EH16 4UU, Great Britain
4. School of Medicine, Kabale University, Box 71 Kabale, Uganda
5. Department of National Disease Control, Ministry of Health, Uganda
6. Directorate of Curative Services, Ministry of Health, Uganda
7. The digital One health Laboratory, Division of Epidemiology Roslin Institute, University of Edinburgh

Keywords: Antibiotic resistance, AMR, Antimicrobial resistance, clinical pathogens, *Staphylococus aureus* in Uganda, GLASS, drug resistance, Covid-19.

Uganda's sentinel site AMR surveillance system

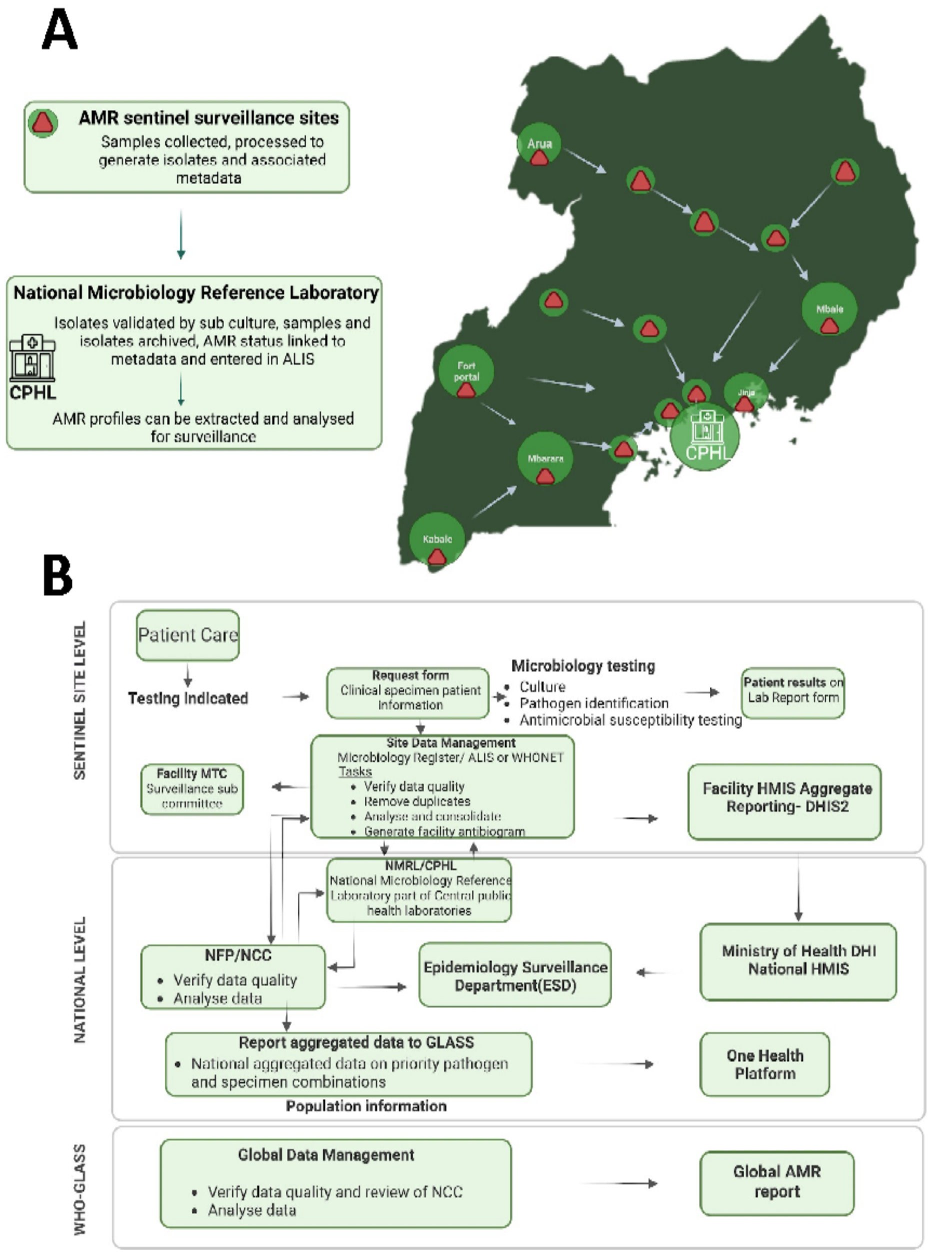

Fig S1: **-** The structure of Uganda's AMR sentinel site surveillance for human health. A- Map of the AMR sentinel surveillance sites and the flow of samples to the National Microbiology Reference Laboratories via the National sample transport network. B- Data flow from AMR sentinel surveillance sites to World health organisation for global reporting. The size of the circle on the map represents the number of isolates collected at a site.

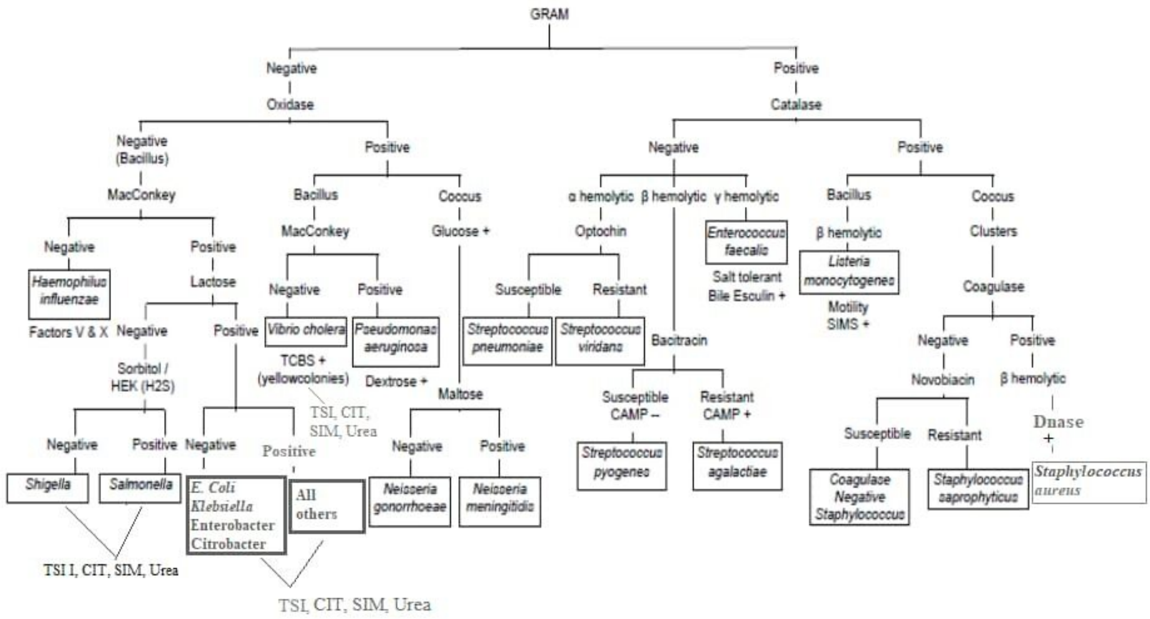

Fig S2: Bacteria identification flow chart.

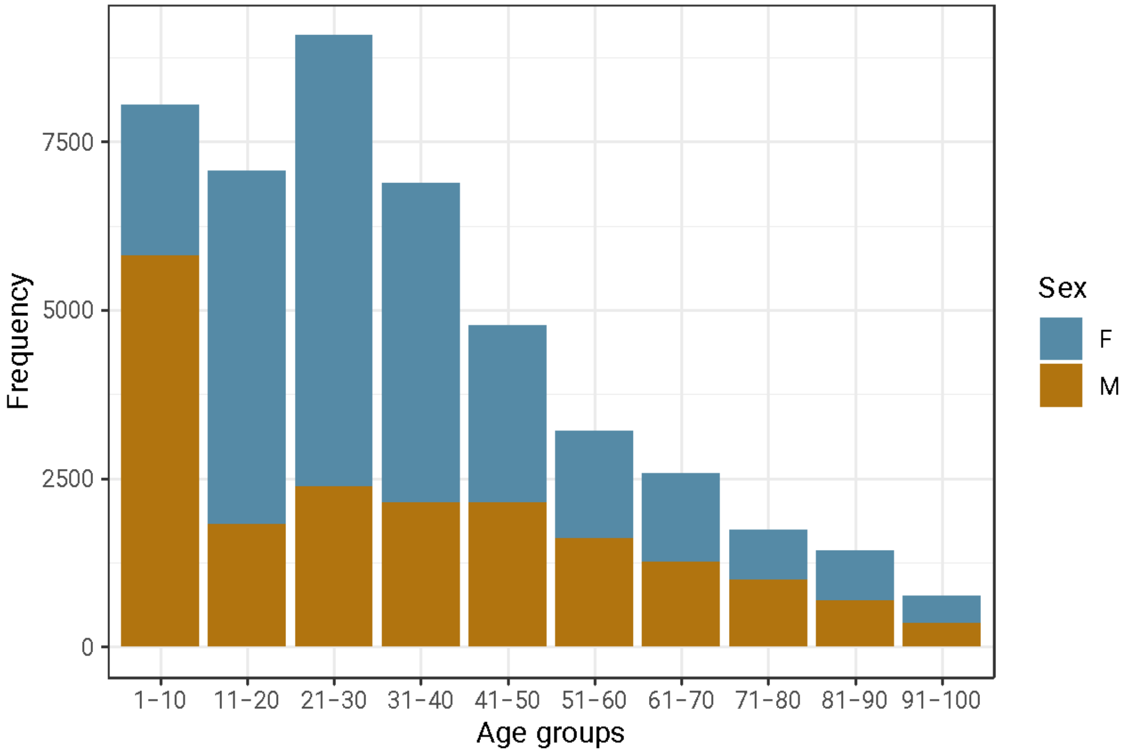

Figure S3: The distribution of sentinel surveillance participants by age and gender.

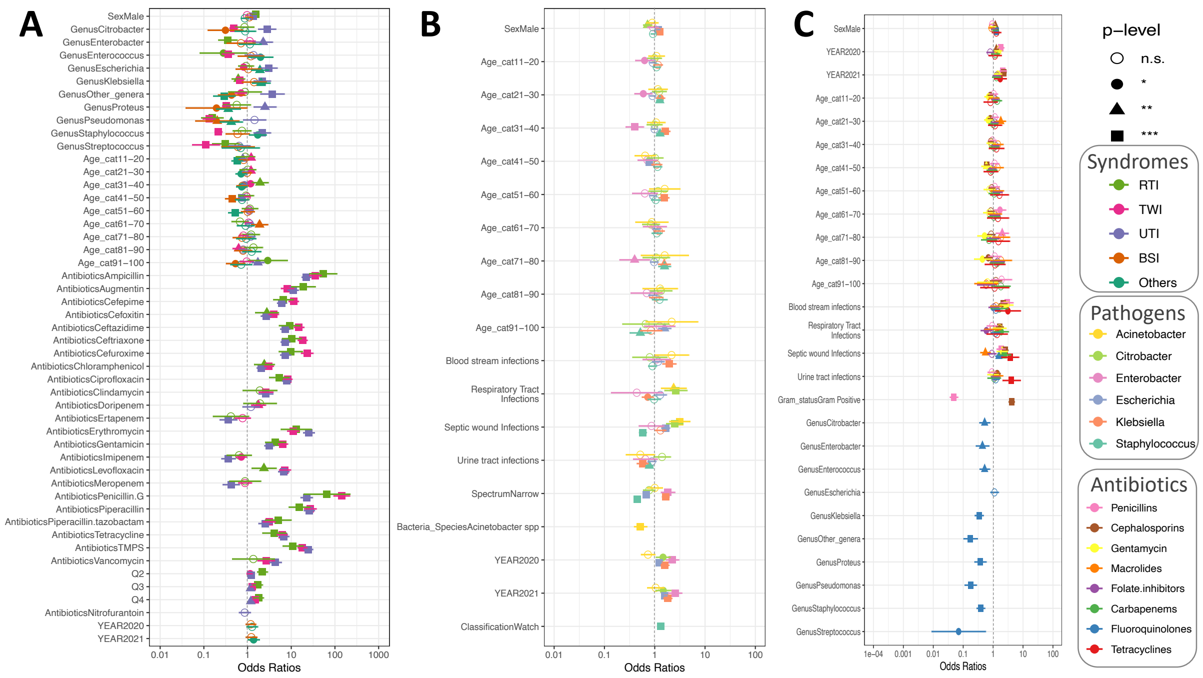

Figure S4. Using a total of 19 mixed effects logistic regression models (R2=10%-54%), we explore factors associated with ABR. Figure 4A compares five models of syndromes, Figure 4B compares six clinical pathogens and Figure 4C compares antibiotic classes. For all three comparisons male patients were more likely to carry ABR pathogens than females. The tabular output of these modes is presented in table S1-S6

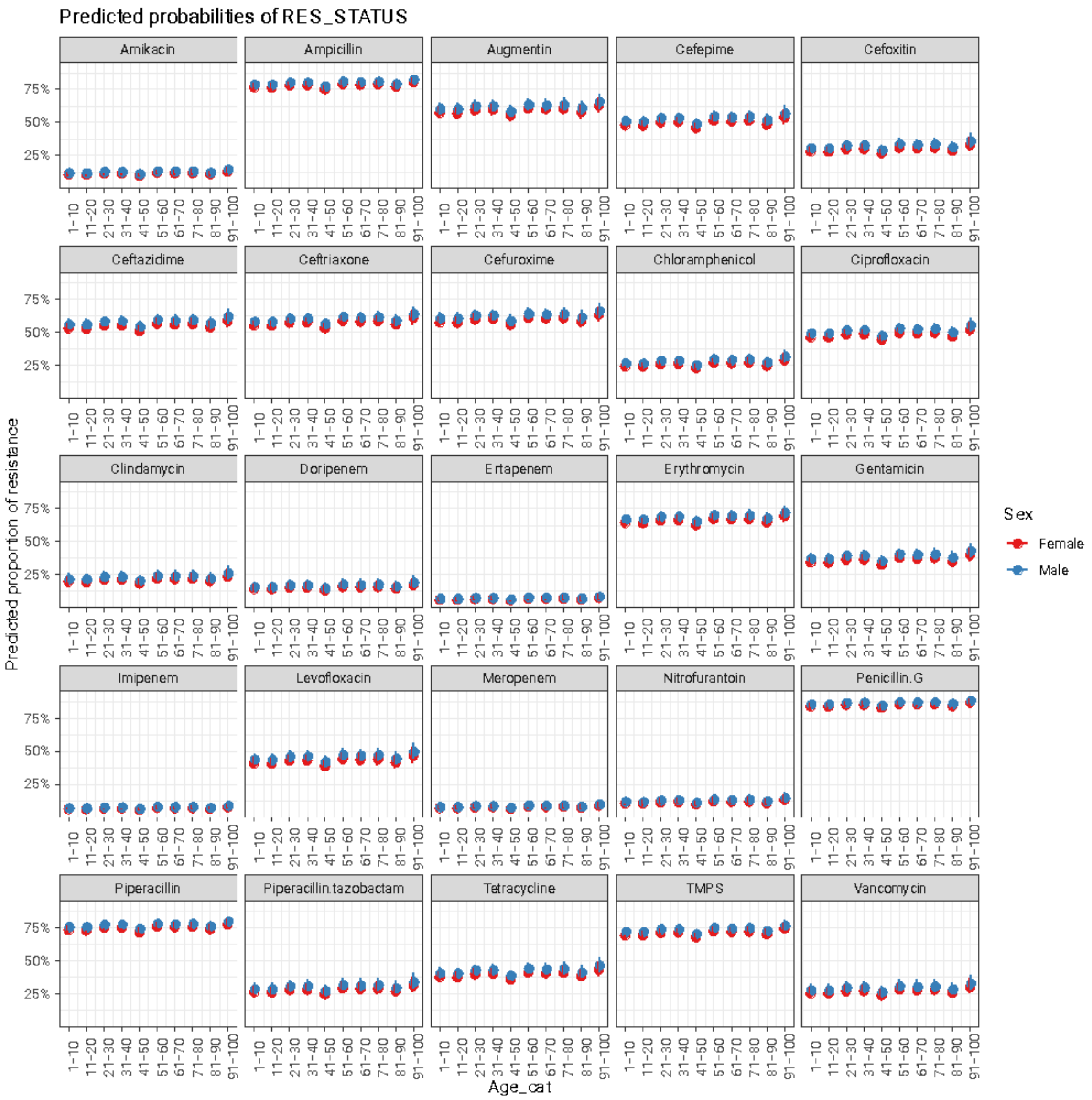

Figure S5: Model predicted prevalence of AMR, visualised by age and predicted resistance coloured by gender and faceted by antibiotic. The estimated ABR is highest and lowest for BSI and RTIs

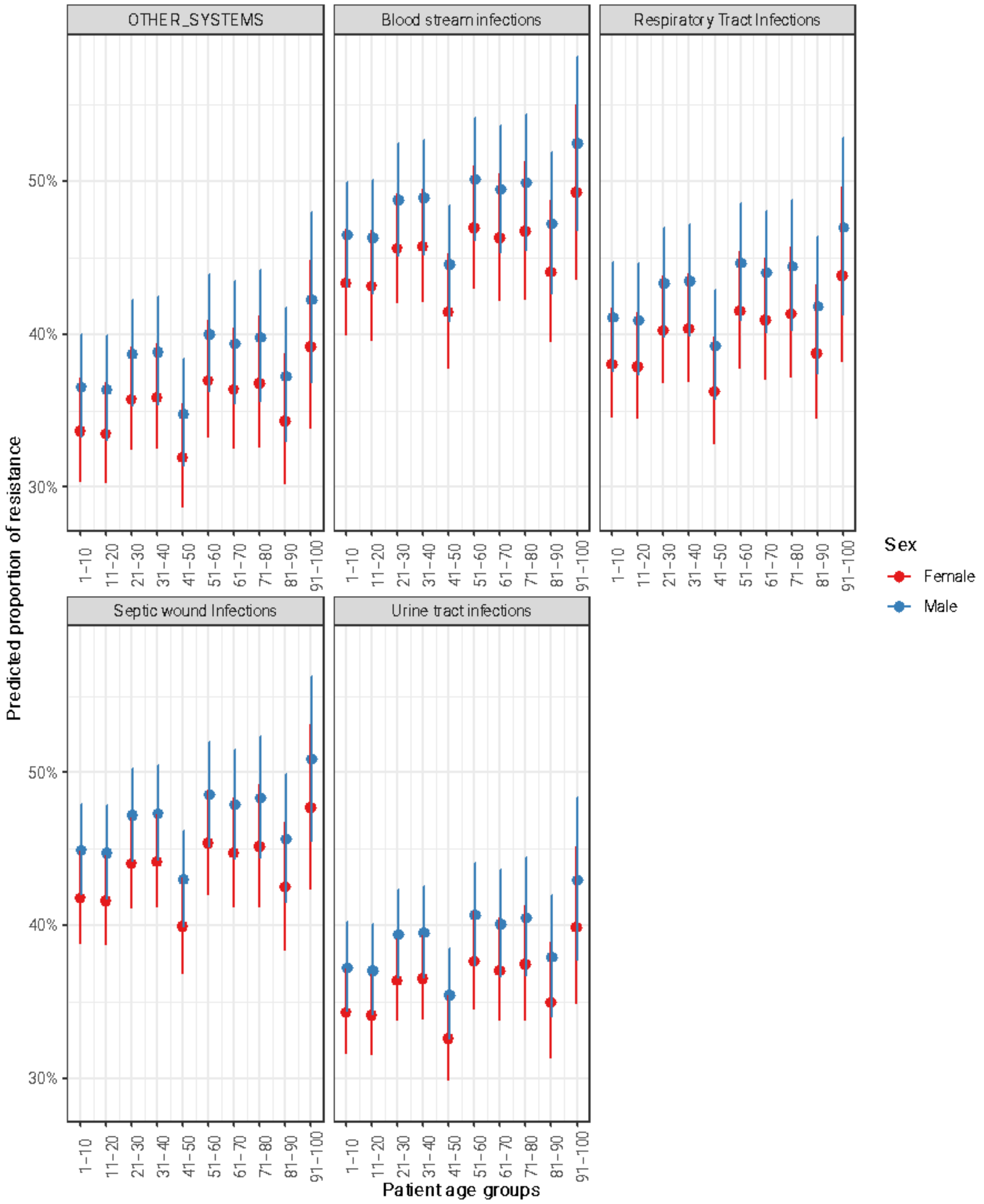

Figure S6: Model predicted prevalence of AMR, visualised by age and predicted resistance coloured by gender and faceted by clinical syndromes. The estimated ABR is highest and lowest for BSI and RTIs

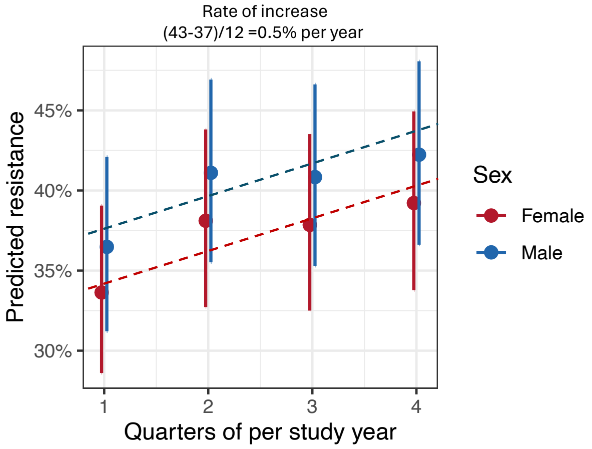

Figure S7: Estimated rate of increase of resistance across the three years of study, this estimate is extracted from a mixed effects model where the year is used as the random variable

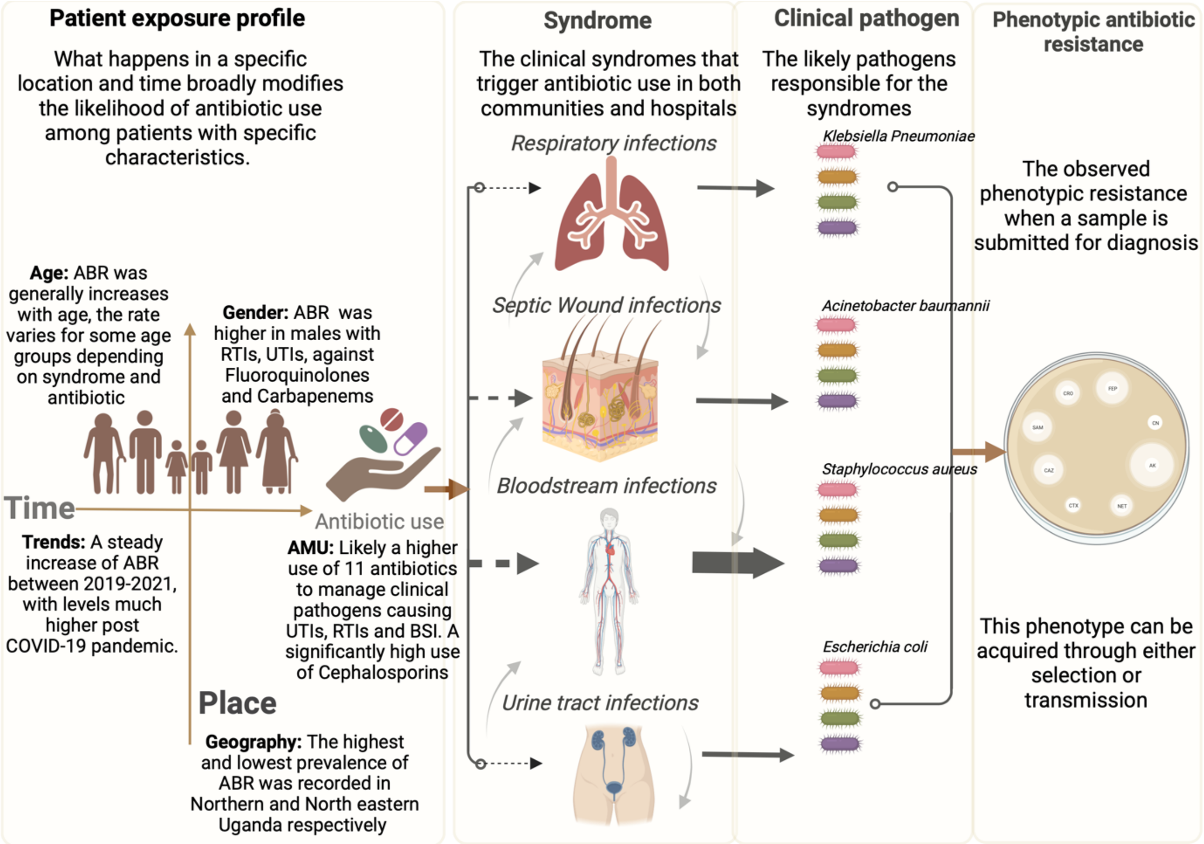

Figure S8: The inferred causal framework of ABR in Uganda based on the modeling output. In Uganda, *K. pneumoniae, A. baumannii, S. aureus*, and *E. coli* were major pathogens observed in the population.

Table S1: The descriptive summary of the AMR sentinel surveillance of Uganda characterising the culture recovery rate as a key diagnostics performance indicator. The bold figures represent the highest and lowest CRR measure per category.

Variable	Levels	Culture recovery			Culture Recovery Rate (CRR) (%)
		Gram- Negative	Gram-Positive	No Growth	
Gender	Male	1543	834	1155	67.2
	Female	2015	1178	2308	58.1
Age	1-10	604	319	269	77.5
	11-20	563	323	349	71.7
	21-30	671	458	985	53.4
	31-40	550	354	987	**47.8**
	41-50	371	209	415	58.2
	51-60	267	121	199	66.0
	61-70	210	96	109	73.7
	71-80	144	61	76	72.9
	81-90	117	45	51	76.0
	91-100	61	26	23	**79.0**
Region	Eastern	274	196	162	74.3
	East Central	165	168	91	78.5
	Central	508	329	2095	**28.5**
	West Nile	177	87	95	73.5
	Western	1812	1097	718	**80.2**
	Mid-Western	591	125	223	76.2
	Northern	17	6	51	31.0
	Northeast	14	6	28	41.6
Year	2019	503	220	1297	**35.7**
	2020	1833	1075	1230	70.2
	2021	1222	717	936	67.4
Syndrome	RTIs	305	59	120	75.2
	BSIs	173	278	276	62.0
	GIT	21	0	29	42.0
	SWIs	1279	631	210	**90.0**
	UTIs	1543	958	2279	53.2
	Neurological	14	5	43	**30.6**
	Others	223	81	506	37.5

Table S2: The output from mixed effects logistic regression model, here we compare two models for each of the gram-positive bacteria using the sjplot package in R

	E. Faecalis		***S. aureus***	
Predictors	*Odds Ratios*	*p*	*Odds Ratios*	*p*
(Intercept)	0.88 (0.66 – 1.16)	0.369	0.62 (0.27 – 1.45)	0.272
Sex [Male]	0.92 (0.83 – 1.02)	0.126	0.92 (0.71 – 1.18)	0.502
Age_cat11-20	1.09 (0.92 – 1.29)	0.330	0.63 * (0.41 – 0.96)	**0.033**
Age_cat21-30	1.26 ** (1.08 – 1.48)	**0.004**	0.59 * (0.39 – 0.89)	**0.012**
Age_cat31-40	1.27 ** (1.07 – 1.51)	**0.007**	0.40 *** (0.26 – 0.60)	**<0.001**
Age_cat41-50	1.14 (0.93 – 1.39)	0.201	0.71 (0.46 – 1.11)	0.136
Age_cat51-60	1.05 (0.84 – 1.30)	0.681	0.63 (0.36 – 1.13)	0.125
Age_cat61-70	1.10 (0.87 – 1.41)	0.425	1.00 (0.58 – 1.72)	0.996
Age_cat71-80	1.57 ** (1.18 – 2.09)	**0.002**	0.40 ** (0.20 – 0.80)	**0.010**
Age_cat81-90	1.25 (0.88 – 1.78)	0.213	0.69 (0.33 – 1.45)	0.328
Age_cat91-100	0.52 ** (0.32 – 0.85)	**0.009**	1.25 (0.62 – 2.54)	0.539
Syndrome [BSI]	0.90 (0.74 – 1.10)	0.294	1.09 (0.58 – 2.06)	0.796
Syndrome [RTI]	0.97 (0.72 – 1.31)	0.852	0.44 (0.13 – 1.43)	0.173
Syndrome [SWI]	0.58 *** (0.48 – 0.69)	**<0.001**	0.86 (0.48 – 1.55)	0.619
Syndrome [UTI]	0.78 ** (0.65 – 0.94)	**0.007**	0.65 (0.37 – 1.13)	0.127
Spectrum [Narrow]	0.45 *** (0.40 – 0.51)	**<0.001**	1.83 *** (1.32 – 2.52)	**<0.001**
Classification [Watch]	1.31 *** (1.18 – 1.45)	**<0.001**		
YEAR [2020]			2.24 *** (1.66 – 3.02)	**<0.001**
YEAR [2021]			2.53 *** (1.85 – 3.48)	**<0.001**
Random Effects				
σ^2	3.29		3.29	
τ_{00}	0.02 YEAR		0.12 Classification	
ICC	0.01		0.04	
N	3 YEAR		2 Classification	
Observations	7279		1982	
Marginal R^2 / Conditional R^2	0.054 / 0.061		0.109 / 0.141	

* *p<0.05*   ** *p<0.01*   *** *p<0.001*

Table S3: The output for mixed effects logistic regression model where the outcome was status of phenotypic resistance (resistance or not resistant). Here we compare between four gram negative clinical pathogens using the sjplot package in R.

	A.baumanni		*C.Fruendii*		*E. coli*		*K. Pneumoniae*	
Predictors	*Odds Ratios*	*p*	*Odds Ratios*	*p*	*Odds Ratios*	*p*	*Odds Ratios*	*p*
(Intercept)	1.01 (0.44 – 2.33)	0.986	0.41 ** (0.21 – 0.78)	**0.006**	0.82 (0.57 – 1.17)	0.274	0.41 (0.16 – 1.07)	0.069
Sex [Male]	0.89 (0.67 – 1.18)	0.409	0.72 ** (0.58 – 0.88)	**0.002**	1.18 *** (1.08 – 1.28)	**<0.001**	1.25 *** (1.10 – 1.42)	**0.001**
Age_cat11-20	1.08 (0.75 – 1.57)	0.674	0.99 (0.71 – 1.37)	0.936	0.91 (0.80 – 1.04)	0.163	1.16 (0.94 – 1.43)	0.175
Age_cat21-30	1.16 (0.75 – 1.79)	0.516	1.23 (0.88 – 1.71)	0.220	0.90 (0.79 – 1.02)	0.097	1.29 * (1.06 – 1.59)	**0.013**
Age_cat31-40	1.07 (0.70 – 1.62)	0.768	0.99 (0.69 – 1.41)	0.946	0.99 (0.87 – 1.13)	0.861	1.63 *** (1.32 – 2.02)	**<0.001**
Age_cat41-50	0.64 (0.40 – 1.04)	0.074	1.01 (0.70 – 1.47)	0.948	0.78 *** (0.68 – 0.90)	**0.001**	1.09 (0.86 – 1.39)	0.483
Age_cat51-60	1.58 (0.78 – 3.19)	0.206	1.16 (0.75 – 1.80)	0.505	0.92 (0.79 – 1.08)	0.323	1.53 *** (1.21 – 1.93)	**<0.001**
Age_cat61-70	0.87 (0.41 – 1.86)	0.726	0.84 (0.56 – 1.25)	0.389	1.08 (0.90 – 1.30)	0.385	1.21 (0.94 – 1.56)	0.130
Age_cat71-80	1.58 (0.53 – 4.69)	0.413	1.07 (0.57 – 1.98)	0.839	0.97 (0.80 – 1.18)	0.775	1.55 ** (1.16 – 2.06)	**0.003**
Age_cat81-90	1.28 (0.57 – 2.86)	0.553	1.33 (0.80 – 2.21)	0.268	0.92 (0.75 – 1.14)	0.471	1.06 (0.77 – 1.47)	0.705
Age_cat91-100	2.17 (0.65 – 7.22)	0.205	0.66 (0.23 – 1.92)	0.446	1.57 ** (1.18 – 2.11)	**0.002**	0.84 (0.51 – 1.39)	0.506
Syndrome [Blood stream infections]	2.16 (0.98 – 4.77)	0.056	0.79 (0.36 – 1.76)	0.570	1.17 (0.88 – 1.55)	0.288	1.95 *** (1.42 – 2.67)	**<0.001**
Syndrome [Respiratory Tract Infections]	2.38 ** (1.33 – 4.26)	**0.004**	2.62 *** (1.56 – 4.42)	**<0.001**	1.29 (0.96 – 1.72)	0.094	0.73 * (0.55 – 0.95)	**0.020**
Syndrome [Septic wound Infections]	3.12 *** (1.94 – 5.02)	**<0.001**	2.48 *** (1.68 – 3.65)	**<0.001**	1.64 *** (1.35 – 2.00)	**<0.001**	1.30 (1.00 – 1.69)	0.050
Syndrome [Urine tract infections]	0.52 (0.27 – 1.00)	0.050	1.40 (0.94 – 2.07)	0.098	0.88 (0.72 – 1.07)	0.195	0.57 *** (0.44 – 0.75)	**<0.001**
Spectrum [Narrow]	1.00 (0.70 – 1.43)	0.999	0.77 (0.58 – 1.02)	0.072	0.68 *** (0.61 – 0.76)	**<0.001**	1.64 *** (1.39 – 1.94)	**<0.001**
Bacteria Species [Acinetobacter spp]	0.52 *** (0.39 – 0.70)	**<0.001**						
YEAR [2020]	0.73 (0.53 – 1.01)	0.059	1.46 * (1.05 – 2.04)	**0.025**	1.23 *** (1.10 – 1.38)	**<0.001**	1.60 *** (1.31 – 1.94)	**<0.001**
YEAR [2021]	1.04 (0.71 – 1.53)	0.837	1.48 * (1.03 – 2.12)	**0.033**	1.57 *** (1.40 – 1.77)	**<0.001**	1.80 *** (1.47 – 2.21)	**<0.001**
Random Effects								
σ^2	3.29		3.29		3.29		3.29	
τ_{00}	0.15 Classification		0.08 Classification		0.04 Classification		0.41 Classification	
ICC	0.04		0.02		0.01		0.11	
N	2 Classification		2 Classification		2 Classification		2 Classification	
Observations	1310		1974		13990		5668	
Marginal R^2 / Conditional R^2	0.114 / 0.153		0.043 / 0.065		0.051 / 0.062		0.072 / 0.175	

* *p<0.05* ** *p<0.01* *** *p<0.001*

Table S4. Summary of culture recovery rate across the 3 years

Organism	*Years* 2019	2020	2021	***Total***
Acinetobacter baumannii	11 (20 %)	34 (61.8 %)	10 (18.2 %)	55 (100 %)
Acinetobacter spp	36 (19.9 %)	92 (50.8 %)	53 (29.3 %)	181 (100 %)
Beta- Hemolytic streptococcus	12 (10.6 %)	67 (59.3 %)	34 (30.1 %)	113 (100 %)
Candida albicans	159 (31.1 %)	210 (41.1 %)	142 (27.8 %)	511 (100 %)
Candida spp	46 (22.2 %)	92 (44.4 %)	69 (33.3 %)	207 (100 %)
Citrobacter freundii	24 (10.5 %)	139 (61 %)	65 (28.5 %)	228 (100 %)
Citrobacter spp	3 (5.6 %)	29 (53.7 %)	22 (40.7 %)	54 (100 %)
Coagulase-negative *Staphylococcus*	82 (9.7 %)	417 (49.5 %)	344 (40.8 %)	843 (100 %)
Cryptococcus spp	2 (11.8 %)	8 (47.1 %)	7 (41.2 %)	17 (100 %)
Enterobacter spp	14 (9.9 %)	63 (44.7 %)	64 (45.4 %)	141 (100 %)
Enterococcus faecalis	64 (14.3 %)	244 (54.5 %)	140 (31.2 %)	448 (100 %)
Enterococcus spp	7 (18.4 %)	14 (36.8 %)	17 (44.7 %)	38 100 %)
Escherichia coli	205 (12.5 %)	849 (51.8 %)	584 (35.7 %)	1638 (100 %)
Klebsiella pneumoniae	62 (10.1 %)	336 (54.9 %)	214 (35 %)	612 (100 %)
Klebsiella spp	15 (18.3 %)	39 (47.6 %)	28 (34.1 %)	82 (100 %)
Mixed Bacterial Growth	8 (44.4 %)	4 (22.2 %)	6 (33.3 %)	18 (100 %)

Morganella morganii	3<br>(12.5 %)	13<br>(54.2 %)	8<br>(33.3 %)	24<br>(100 %)
Neisseria gonorrhoeae	0<br>(0 %)	0<br>(0 %)	34<br>(100 %)	34<br>(100 %)
No growth	1065<br>(41.1 %)	849<br>(32.8 %)	677<br>(26.1 %)	2591<br>(100 %)
Proteus spp	43<br>(26.1 %)	73<br>(44.2 %)	49<br>(29.7 %)	165<br>(100 %)
Providencia spp	0<br>(0 %)	13<br>(59.1 %)	9<br>(40.9 %)	22<br>(100 %)
Pseudomonas aeruginosa	31<br>(20.7 %)	74<br>(49.3 %)	45<br>(30 %)	150<br>(100 %)
Pseudomonas spp	4<br>(14.3 %)	11<br>(39.3 %)	13<br>(46.4 %)	28<br>(100 %)
Salmonella spp	19<br>(30.6 %)	36<br>(58.1 %)	7<br>(11.3 %)	62<br>(100 %)
Serratia spp	3<br>(21.4 %)	5<br>(35.7 %)	6<br>(42.9 %)	14<br>(100 %)
Shigella spp	6<br>(35.3 %)	11<br>(64.7 %)	0<br>(0 %)	17<br>(100 %)
Staphylococcus aureus	57<br>(8.5 %)	396<br>(59.4 %)	214<br>(32.1 %)	667<br>(100 %)
Staphylococcus saprophyticus	6<br>(75 %)	2<br>(25 %)	0<br>(0 %)	8<br>(100 %)
Streptococcus pneumoniae	3<br>(42.9 %)	2<br>(28.6 %)	2<br>(28.6 %)	7<br>(100 %)
Vibrio cholerae	22<br>(44.9 %)	16<br>(32.7 %)	11<br>(22.4 %)	49<br>(100 %)
**Total**	**2012<br>(22.3 %)**	**4138<br>(45.9 %)**	**2874<br>(31.8 %)**	**9024<br>(100 %)**

χ^2=1108.281 · df=58 · Cramer's V=0.248 · Fisher's p=0.000

Table S5:The output for mixed effects logistic regression model where the outcome was status of phenotypic resistance (resistance or not resistant). Here we compare between four major antibiotic classes using the sjplot package in R

	Beta lactams		Penicillin		Cephalosporins		Carbapenems	
Predictors	*Odds Ratios*	*p*	*Odds Ratios*	*p*	*Odds Ratios*	*p*	*Odds Ratios*	*p*
(Intercept)	0.48 (0.18 – 1.28)	0.144	5.17 * (1.09 – 24.59)	**0.039**	0.38 * (0.16 – 0.87)	**0.023**	0.05 *** (0.02 – 0.10)	**<0.001**
Sex [Male]	0.74 * (0.56 – 0.97)	**0.031**	0.93 (0.77 – 1.12)	0.435	1.19 ** (1.05 – 1.34)	**0.006**	1.20 (0.96 – 1.51)	0.117
YEAR [2020]	5.68 *** (3.95 – 8.17)	**<0.001**	1.80 *** (1.40 – 2.31)	**<0.001**	1.28 ** (1.07 – 1.53)	**0.006**	1.19 (0.86 – 1.65)	0.301
YEAR [2021]	1.79 ** (1.25 – 2.55)	**0.001**	2.14 *** (1.63 – 2.81)	**<0.001**	2.27 *** (1.88 – 2.75)	**<0.001**	1.31 (0.92 – 1.85)	0.132
Age_cat11-20	0.86 (0.55 – 1.35)	0.513	1.05 (0.77 – 1.43)	0.778	0.80 * (0.66 – 0.97)	**0.024**	1.37 (0.96 – 1.94)	0.079
Age_cat21-30	0.93 (0.62 – 1.39)	0.713	1.14 (0.85 – 1.53)	0.384	0.80 * (0.67 – 0.97)	**0.024**	1.00 (0.70 – 1.44)	0.985
Age_cat31-40	1.47 (0.95 – 2.27)	0.087	0.95 (0.70 – 1.28)	0.724	0.84 (0.69 – 1.03)	0.087	1.07 (0.74 – 1.56)	0.721
Age_cat41-50	1.09 (0.68 – 1.73)	0.723	1.12 (0.80 – 1.57)	0.509	0.61 *** (0.49 – 0.76)	**<0.001**	0.90 (0.60 – 1.35)	0.625
Age_cat51-60	1.65 * (1.00 – 2.71)	**0.049**	1.13 (0.77 – 1.64)	0.541	0.90 (0.70 – 1.15)	0.390	1.09 (0.71 – 1.66)	0.705
Age_cat61-70	1.24 (0.69 – 2.23)	0.472	1.70 * (1.08 – 2.67)	**0.023**	0.84 (0.65 – 1.08)	0.173	1.06 (0.67 – 1.69)	0.807
Age_cat71-80	1.63 (0.85 – 3.13)	0.141	2.02 ** (1.22 – 3.32)	**0.006**	0.86 (0.63 – 1.17)	0.341	0.73 (0.40 – 1.33)	0.303
Age_cat81-90	1.62 (0.82 – 3.22)	0.167	1.13 (0.69 – 1.86)	0.625	0.69 * (0.50 – 0.95)	**0.022**	1.34 (0.77 – 2.35)	0.301
Age_cat91-100	2.21 (0.75 – 6.47)	0.149	1.92 (0.88 – 4.21)	0.103	1.23 (0.78 – 1.96)	0.371	1.81 (0.87 – 3.77)	0.110
Syndrome [Blood stream infections]	1.42 (0.65 – 3.09)	0.380	2.96 *** (1.85 – 4.72)	**<0.001**	2.15 *** (1.55 – 2.96)	**<0.001**	1.62 (0.82 – 3.18)	0.165
Syndrome [Respiratory Tract Infections]	1.14 (0.59 – 2.21)	0.700	0.93 (0.61 – 1.41)	0.738	1.65 *** (1.23 – 2.22)	**0.001**	1.86 * (1.05 – 3.30)	**0.034**
Syndrome [Septic wound Infections]	0.85 (0.47 – 1.53)	0.592	1.84 *** (1.31 – 2.58)	**<0.001**	2.52 *** (1.98 – 3.20)	**<0.001**	2.40 *** (1.48 – 3.91)	**<0.001**
Syndrome [Urine tract infections]	0.66 (0.37 – 1.20)	0.173	0.91 (0.65 – 1.26)	0.555	1.37 * (1.07 – 1.75)	**0.012**	1.07 (0.65 – 1.78)	0.787
Gram status [Gram Positive]			0.05 *** (0.04 – 0.07)	**<0.001**	4.14 *** (3.29 – 5.21)	**<0.001**		

Random Effects				
σ^2	3.29	3.29	3.29	3.29
τ_{00}	0.25 $_{Antibiotics}$	1.74 $_{Antibiotics}$	0.63 $_{Antibiotics}$	0.16 $_{Antibiotics}$
ICC	0.07	0.35	0.16	0.05
N	2 $_{Antibiotics}$	3 $_{Antibiotics}$	4 $_{Antibiotics}$	4 $_{Antibiotics}$
Observations	1393	4134	6129	4601
Marginal R^2 / Conditional R^2	0.140 / 0.200	0.298 / 0.541	0.103 / 0.246	0.054 / 0.100

* $p<0.05$ ** $p<0.01$ *** $p<0.001$

Table S6: The output for mixed effects logistic regression model where the outcome was status of phenotypic resistance (resistance or not resistant). Here we compare between other antibiotic classes using the sjplot package in R. Here in some of the classes we had one antibiotic represented.

	Gentamycin		Erythromycin		TMPS		Fluoroquinolones		Tetracyclines	
Predictors	*Odds Ratios*	*p*	*Odds Ratios*	*p*	*Odds Ratios*	*p*	*Odds Ratios*	*p*	*Odds Ratios*	*p*
(Intercept)	0.33 ** (0.16 – 0.67)	**0.002**	0.94 (0.54 – 1.63)	0.833	2.20 ** (1.35 – 3.58)	**0.001**	1.05 (0.71 – 1.56)	0.797	0.04 * (0.00 – 0.98)	**0.049**
Sex [Male]	1.00 (0.80 – 1.23)	0.969	0.88 (0.70 – 1.12)	0.293	1.07 (0.89 – 1.29)	0.485	1.28 *** (1.12 – 1.48)	**<0.001**	1.29 (0.91 – 1.82)	0.157
YEAR [2020]	1.46 ** (1.10 – 1.94)	**0.009**			0.80 (0.60 – 1.06)	0.120			1.16 (0.70 – 1.90)	0.565
YEAR [2021]	1.44 * (1.07 – 1.95)	**0.017**			1.31 (0.96 – 1.79)	0.084			1.73 * (1.07 – 2.79)	**0.024**
Age_cat11-20	0.71 * (0.51 – 0.98)	**0.040**	1.06 (0.72 – 1.58)	0.757	1.16 (0.87 – 1.55)	0.304			0.82 (0.48 – 1.39)	0.455
Age_cat21-30	0.69 * (0.50 – 0.94)	**0.020**	1.81 ** (1.24 – 2.63)	**0.002**	1.13 (0.85 – 1.50)	0.385			1.15 (0.68 – 1.96)	0.593
Age_cat31-40	0.84 (0.61 – 1.17)	0.298	1.46 (0.98 – 2.18)	0.065	1.20 (0.88 – 1.62)	0.245			1.29 (0.72 – 2.30)	0.394
Age_cat41-50	0.59 ** (0.40 – 0.87)	**0.008**	1.41 (0.90 – 2.21)	0.136	0.93 (0.67 – 1.27)	0.637			0.83 (0.48 – 1.44)	0.515
Age_cat51-60	0.75 (0.49 – 1.17)	0.205	1.36 (0.82 – 2.26)	0.234	1.40 (0.95 – 2.04)	0.087			1.63 (0.80 – 3.31)	0.177
Age_cat61-70	0.73 (0.47 – 1.15)	0.179	1.13 (0.66 – 1.93)	0.659	1.14 (0.76 – 1.71)	0.518			1.45 (0.63 – 3.34)	0.381
Age_cat71-80	0.52 * (0.30 – 0.90)	**0.019**	1.83 (0.93 – 3.59)	0.079	0.99 (0.62 – 1.58)	0.961			1.51 (0.63 – 3.60)	0.358
Age_cat81-90	0.44 * (0.23 – 0.84)	**0.013**	1.84 (0.81 – 4.19)	0.146	1.40 (0.81 – 2.42)	0.230			1.16 (0.53 – 2.55)	0.715
Age_cat91-100	0.64 (0.29 – 1.41)	0.270	0.59 (0.24 – 1.47)	0.256	0.58 (0.29 – 1.16)	0.124			1.12 (0.37 – 3.38)	0.836
Syndrome [BSI]	2.79 *** (1.67 – 4.67)	**<0.001**	1.24 (0.79 – 1.94)	0.344	1.06 (0.70 – 1.60)	0.785	1.23 (0.88 – 1.74)	0.228	3.12 * (1.17 – 8.31)	**0.023**
Syndrome [RTI	1.64 (0.98 – 2.75)	0.059	0.96 (0.50 – 1.83)	0.904	0.60 * (0.38 – 0.92)	**0.021**	1.18 (0.82 – 1.69)	0.364	1.35 (0.61 – 3.01)	0.463
Syndrome SWI]	2.00 ** (1.31 – 3.04)	**0.001**	0.55 ** (0.37 – 0.81)	**0.003**	0.91 (0.65 – 1.27)	0.581	1.56 ** (1.19 – 2.05)	**0.001**	3.68 *** (1.87 – 7.25)	**<0.001**
Syndrome [UTI]	0.98 (0.64 – 1.51)	0.945	1.43 (0.97 – 2.12)	0.071	1.19 (0.85 – 1.67)	0.318	1.23 (0.92 – 1.62)	0.157	4.11 *** (2.06 – 8.20)	**<0.001**
Genus [Citrobacter]							0.52 ** (0.35 – 0.78)	**0.002**		

Genus [Enterobacter]				0.44 ** (0.26 – 0.73)	**0.002**	
Genus [Enterococcus]				0.52 ** (0.35 – 0.78)	**0.001**	
Genus [Escherichia]				1.11 (0.81 – 1.53)	0.513	
Genus [Klebsiella]				0.34 *** (0.24 – 0.48)	**<0.001**	
Genus [Other_genera]				0.17 *** (0.10 – 0.30)	**<0.001**	
Genus [Proteus]				0.37 *** (0.24 – 0.59)	**<0.001**	
Genus [Pseudomonas]				0.18 *** (0.11 – 0.28)	**<0.001**	
Genus [Staphylococcus]				0.38 *** (0.28 – 0.53)	**<0.001**	
Genus [Streptococcus]				0.07 * (0.01 – 0.56)	**0.012**	
Random Effects						
σ^2	3.29	3.29	3.29	3.29		3.29
τ_{00}	0.11 $_{Gram\_status}$	0.06 $_{YEAR}$	0.02 $_{Gram\_status}$	0.00 $_{Antibiotics}$		5.29 $_{Gram\_status}$
ICC	0.03	0.02	0.01			0.62
N	2 $_{Gram\_status}$	3 $_{YEAR}$	2 $_{Gram\_status}$	2 $_{Antibiotics}$		2 $_{Gram\_status}$
Observations	2031	1423	2848	4212		1270
Marginal R^2 / Conditional R^2	0.064 / 0.093	0.078 / 0.094	0.029 / 0.034	0.107 / NA		0.026 / 0.627

* *p<0.05*   ** *p<0.01*   *** *p<0.001*

Table S7:The output for mixed effects logistic regression model where the outcome was status of phenotypic resistance (resistance or not resistant). Here we compare between clinical syndromes using the sjplot package in R

	RTIs		SWIs		UTIs		BSI		Others	
Predictors	*Odds Ratios*	*P*	*Odds Ratios*	*p*	*Odds Ratios*	*p*	*Odds Ratios*	*p*	*Odds Ratios*	*P*
(Intercept)	0.10 ***<br>(0.05 – 0.21)	**<0.001**	0.19 ***<br>(0.13 – 0.28)	**<0.001**	0.04 ***<br>(0.02 – 0.08)	**<0.001**	1.06<br>(0.51 – 2.19)	0.881	0.57<br>(0.30 – 1.11)	0.098
Male	1.53 ***<br>(1.27 – 1.84)	**<0.001**	0.97<br>(0.89 – 1.07)	0.549	1.36 ***<br>(1.24 – 1.49)	**<0.001**	1.10<br>(0.93 – 1.29)	0.265	0.86<br>(0.72 – 1.04)	0.127
Citrobacter	0.85<br>(0.51 – 1.41)	0.527	0.48 ***<br>(0.38 – 0.59)	**<0.001**	2.79 ***<br>(1.74 – 4.46)	**<0.001**	0.31 *<br>(0.12 – 0.80)	**0.015**	0.89<br>(0.51 – 1.55)	0.669
Enterobacter	0.35 ***<br>(0.21 – 0.58)	**<0.001**	1.12<br>(0.83 – 1.51)	0.469	2.28 **<br>(1.38 – 3.77)	**0.001**	0.71<br>(0.33 – 1.51)	0.367	1.13<br>(0.62 – 2.06)	0.689
Enterococcus	0.28 *<br>(0.08 – 0.98)	**0.046**	0.36 ***<br>(0.26 – 0.49)	**<0.001**	1.37<br>(0.86 – 2.18)	0.190	1.19<br>(0.60 – 2.39)	0.620	1.98 *<br>(1.02 – 3.83)	**0.043**
Escherichia]	0.92<br>(0.61 – 1.39)	0.692	0.81 *<br>(0.68 – 0.96)	**0.017**	3.09 ***<br>(1.98 – 4.81)	**<0.001**	1.04<br>(0.53 – 2.02)	0.915	1.92 **<br>(1.20 – 3.07)	**0.006**
Klebsiella]	0.61 **<br>(0.43 – 0.87)	**0.006**	0.67 ***<br>(0.55 – 0.81)	**<0.001**	2.16 ***<br>(1.37 – 3.39)	**0.001**	1.41<br>(0.74 – 2.70)	0.296	2.05 **<br>(1.26 – 3.33)	**0.004**
Other_genera	0.87<br>(0.36 – 2.10)	0.754	0.71 *<br>(0.51 – 0.97)	**0.033**	3.69 ***<br>(1.96 – 6.96)	**<0.001**	0.43 *<br>(0.21 – 0.88)	**0.021**	0.29 ***<br>(0.16 – 0.53)	**<0.001**
Proteus	0.57<br>(0.27 – 1.18)	0.129	0.33 ***<br>(0.26 – 0.40)	**<0.001**	2.50 **<br>(1.38 – 4.53)	**0.003**	0.19 *<br>(0.04 – 0.99)	**0.048**	0.36 **<br>(0.19 – 0.69)	**0.002**
Pseudomonas	0.16 ***<br>(0.09 – 0.28)	**<0.001**	0.13 ***<br>(0.09 – 0.18)	**<0.001**	1.44<br>(0.79 – 2.63)	0.234	0.20 **<br>(0.06 – 0.64)	**0.007**	0.42 **<br>(0.23 – 0.78)	**0.005**
Staphylococcus	0.74<br>(0.46 – 1.21)	0.236	0.21 ***<br>(0.17 – 0.26)	**<0.001**	2.15 ***<br>(1.36 – 3.40)	**0.001**	0.59<br>(0.31 – 1.11)	0.103	1.71 *<br>(1.08 – 2.71)	**0.023**
Streptococcus	0.31 ***<br>(0.15 – 0.62)	**0.001**	0.11 ***<br>(0.05 – 0.23)	**<0.001**	0.64<br>(0.35 – 1.15)	0.137	0.60<br>(0.25 – 1.44)	0.254	0.70<br>(0.26 – 1.89)	0.479
Age 11-20	0.89<br>(0.54 – 1.47)	0.651	1.21 **<br>(1.05 – 1.39)	**0.007**	0.78 **<br>(0.66 – 0.92)	**0.003**	0.81<br>(0.62 – 1.07)	0.143	0.58 ***<br>(0.43 – 0.78)	**<0.001**
Age 21-30	1.01<br>(0.65 – 1.56)	0.973	1.20 **<br>(1.05 – 1.38)	**0.009**	0.86<br>(0.74 – 1.00)	0.055	0.97<br>(0.75 – 1.25)	0.823	0.71 *<br>(0.53 – 0.96)	**0.027**
Age 31-40	1.93 **<br>(1.24 – 3.00)	**0.003**	1.17 *<br>(1.01 – 1.36)	**0.034**	0.85 *<br>(0.72 – 1.00)	**0.047**	0.76<br>(0.55 – 1.05)	0.099	0.73 *<br>(0.55 – 0.99)	**0.042**
Age 41-50	0.92<br>(0.61 – 1.39)	0.693	0.96<br>(0.82 – 1.13)	0.647	0.74 ***<br>(0.63 – 0.88)	**0.001**	0.44 ***<br>(0.30 – 0.65)	**<0.001**	0.75<br>(0.52 – 1.07)	0.114
Age 51-60	1.13<br>(0.74 – 1.73)	0.571	1.08<br>(0.91 – 1.28)	0.380	1.11<br>(0.91 – 1.35)	0.313	1.01<br>(0.71 – 1.44)	0.964	0.53 ***<br>(0.36 – 0.77)	**0.001**
Age 61-70	0.67<br>(0.43 – 1.05)	0.078	1.05<br>(0.88 – 1.27)	0.580	1.12<br>(0.91 – 1.38)	0.293	1.88 **<br>(1.21 – 2.92)	**0.005**	0.90<br>(0.57 – 1.43)	0.654
Age 71-80	1.21<br>(0.77 – 1.90)	0.407	0.80<br>(0.63 – 1.02)	0.074	1.19<br>(0.94 – 1.50)	0.153	0.74<br>(0.45 – 1.22)	0.241	0.93<br>(0.55 – 1.56)	0.777
Age 81-90	1.36<br>(0.84 – 2.23)	0.215	0.63 **<br>(0.45 – 0.87)	**0.005**	0.80<br>(0.63 – 1.00)	0.054	0.79<br>(0.50 – 1.25)	0.318	1.24<br>(0.76 – 2.03)	0.392
Age 91-100	2.90 *<br>(1.02 – 8.19)	**0.045**	0.96<br>(0.66 – 1.37)	0.808	1.72 **<br>(1.23 – 2.41)	**0.001**	0.53 *<br>(0.32 – 0.87)	**0.013**	0.71<br>(0.40 – 1.26)	0.246

Ampicillin	53.35 *** (25.62 – 111.08)	**<0.001**	35.50 *** (25.76 – 48.92)	**<0.001**	22.27 *** (17.20 – 28.85)	**<0.001**
Augmentin	18.80 *** (9.89 – 35.75)	**<0.001**	8.15 *** (5.79 – 11.48)	**<0.001**	10.97 *** (8.18 – 14.72)	**<0.001**
Cefepime	6.60 *** (3.86 – 11.29)	**<0.001**	11.30 *** (8.68 – 14.70)	**<0.001**	6.09 *** (4.71 – 7.87)	**<0.001**
Cefoxitin	2.77 ** (1.49 – 5.15)	**0.001**	3.94 *** (3.01 – 5.15)	**<0.001**	2.65 *** (2.00 – 3.50)	**<0.001**
Ceftazidime	9.13 *** (5.20 – 16.01)	**<0.001**	15.03 *** (11.51 – 19.63)	**<0.001**	7.24 *** (5.57 – 9.40)	**<0.001**
Ceftriaxone	10.30 *** (6.16 – 17.24)	**<0.001**	18.15 *** (14.09 – 23.38)	**<0.001**	7.30 *** (5.72 – 9.30)	**<0.001**
Cefuroxime	9.68 *** (5.16 – 18.15)	**<0.001**	23.03 *** (16.66 – 31.86)	**<0.001**	7.36 *** (5.54 – 9.78)	**<0.001**
Chloramphenicol	2.41 ** (1.40 – 4.16)	**0.001**	3.06 *** (2.38 – 3.94)	**<0.001**	2.08 *** (1.61 – 2.68)	**<0.001**
Ciprofloxacin	5.26 *** (3.10 – 8.95)	**<0.001**	8.27 *** (6.50 – 10.51)	**<0.001**	7.87 *** (6.19 – 10.00)	**<0.001**
Clindamycin	1.91 (0.77 – 4.73)	0.162	2.63 *** (1.79 – 3.86)	**<0.001**	2.62 *** (1.80 – 3.83)	**<0.001**
Doripenem	1.92 (0.80 – 4.61)	0.145	1.77 ** (1.21 – 2.60)	**0.003**	1.20 (0.76 – 1.88)	0.436
Ertapenem	0.41 (0.16 – 1.07)	0.069	0.77 (0.51 – 1.17)	0.222	0.36 *** (0.23 – 0.58)	**<0.001**
Erythromycin	12.87 *** (5.75 – 28.81)	**<0.001**	10.99 *** (8.07 – 14.97)	**<0.001**	25.22 *** (18.68 – 34.06)	**<0.001**
Gentamicin	4.36 *** (2.50 – 7.57)	**<0.001**	6.44 *** (4.97 – 8.33)	**<0.001**	3.14 *** (2.41 – 4.08)	**<0.001**
Imipenem	0.64 (0.33 – 1.23)	0.182	0.72 * (0.54 – 0.96)	**0.028**	0.36 *** (0.25 – 0.53)	**<0.001**
Levofloxacin	2.37 ** (1.23 – 4.56)	**0.010**	7.15 *** (5.25 – 9.75)	**<0.001**	6.69 *** (4.98 – 8.99)	**<0.001**
Meropenem	0.88 (0.38 – 2.02)	0.757	0.87 (0.61 – 1.24)	0.453	0.42 *** (0.27 – 0.66)	**<0.001**
Penicillin.G	65.33 *** (19.20 – 222.24)	**<0.001**	142.39 *** (92.54 – 219.08)	**<0.001**	22.37 *** (16.24 – 30.83)	**<0.001**
Piperacillin	15.32 *** (8.74 – 26.85)	**<0.001**	27.14 *** (19.61 – 37.56)	**<0.001**	25.76 *** (19.41 – 34.18)	**<0.001**
Piperacillin.tazobactam	5.01 *** (2.50 – 10.02)	**<0.001**	3.10 *** (2.23 – 4.33)	**<0.001**	2.55 *** (1.77 – 3.68)	**<0.001**
Tetracycline	4.10 *** (2.18 – 7.71)	**<0.001**	6.16 *** (4.66 – 8.15)	**<0.001**	6.76 *** (5.12 – 8.93)	**<0.001**
TMPS	10.94 *** (6.36 – 18.82)	**<0.001**	17.95 *** (13.98 – 23.06)	**<0.001**	24.28 *** (18.86 – 31.27)	**<0.001**
Vancomycin	1.35 (0.44 – 4.13)	0.595	2.65 *** (1.68 – 4.17)	**<0.001**	4.34 *** (3.15 – 5.99)	**<0.001**
Q2	2.18 *** (1.65 – 2.90)	**<0.001**	1.15 * (1.02 – 1.29)	**0.020**	1.23 *** (1.11 – 1.36)	**<0.001**

Q3	1.72 *** (1.33 – 2.22)	**<0.001**	1.29 *** (1.15 – 1.44)	**<0.001**	1.17 ** (1.06 – 1.29)	**0.002**				
Q4	1.78 *** (1.36 – 2.34)	**<0.001**	1.50 *** (1.32 – 1.70)	**<0.001**	1.19 ** (1.07 – 1.32)	**0.002**				
Nitrofurantoin					0.86 (0.63 – 1.17)	0.343				
YEAR 2020							1.18 (0.90 – 1.55)	0.227	1.27 (0.94 – 1.71)	0.119
YEAR 2021							1.22 (0.91 – 1.64)	0.191	1.38 * (1.01 – 1.88)	**0.044**
Random Effects										
σ^2	3.29		3.29		3.29		3.29		3.29	
τ_{00}	0.02 $_{YEAR}$		0.05 $_{YEAR}$		0.07 $_{YEAR}$		0.06 $_{Classification}$		0.09 $_{Classification}$	
ICC	0.01		0.01		0.02		0.02		0.03	
N	3 $_{YEAR}$		3 $_{YEAR}$		3 $_{YEAR}$		2 $_{Classification}$		2 $_{Classification}$	
Observations	2819		13517		16746		2644		2435	
Marginal R^2 / Conditional R^2	0.351 / 0.355		0.353 / 0.363		0.341 / 0.354		0.078 / 0.094		0.150 / 0.172	

* *p<0.05* ** *p<0.01* *** *p<0.001*
